# Self-Assembly of Amphiphilic Dendrimers: The Role of Generation and Alkyl Chain Length in siRNA Interaction

**DOI:** 10.1038/srep29436

**Published:** 2016-07-05

**Authors:** Valeria Márquez-Miranda, Ingrid Araya-Durán, María Belén Camarada, Jeffrey Comer, Jesús A. Valencia-Gallegos, Fernando Danilo González-Nilo

**Affiliations:** 1 Universidad Andres Bello, Facultad de Biología, Center for Bioinformatics and Integrative Biology (CBIB), Av. República 239, Santiago, Chile; 2 Fundación Fraunhofer Chile Research, Las Condes, Chile; 3 Universidad Bernardo O Higgins, Laboratorio de Bionanotecnología, General Gana 1702, Santiago, Chile; 4 Kansas State University, Nanotechnology Innovation Center of Kansas State, Institute of Computational Comparative Medicine, Anatomy and Physiology, Kansas, USA; 5 Centro de Biotecnología FEMSA, Escuela de Ingeniería y Ciencias, Tecnológico de Monterrey, Av. Eugenio Garza Sada 2501 Sur, Col. Tecnológico, Monterrey, N.L, México; 6 Centro Interdisciplinario de Neurociencia de Valparaíso, Facultad de Ciencias, Universidad de Valparaíso, Valparaíso, Chile

## Abstract

An ideal nucleic-acid transfection system should combine the physical and chemical characteristics of cationic lipids and linear polymers to decrease cytotoxicity and uptake limitations. Previous research described new types of carriers termed amphiphilic dendrimers (ADs), which are based on polyamidoamine dendrimers (PAMAM). These ADs display the cell membrane affinity advantage of lipids and preserve the high affinity for DNA possessed by cationic dendrimers. These lipid/dendrimer hybrids consist of a low-generation, hydrophilic dendron (G2, G1, or G0) bonded to a hydrophobic tail. The G2-18C AD was reported to be an efficient siRNA vector with significant gene silencing. However, shorter tail ADs (G2-15C and G2-13C) and lower generation (G0 and G1) dendrimers failed as transfection carriers. To date, the self-assembly phenomenon of this class of amphiphilic dendrimers has not been molecularly explored using molecular simulation methods. To gain insight into these systems, the present study used coarse-grained molecular dynamics simulations to describe how ADs are able to self-assemble into an aggregate, and, specifically, how tail length and generation play a key role in this event. Finally, explanations are given for the better efficiency of G2/18-C as gene carrier in terms of binding of siRNA. This knowledge could be relevant for the design of novel, safer ADs with well-optimized affinity for siRNA.

The development of safe and efficient carriers is one of the most important requirements for the clinical implementation of nucleic acid-based therapies. Of the non-viral delivery systems, cationic lipid and polymer members are the most popular^1^. Liposomes composed of cationic lipids are successful systems for DNA delivery. However, there are many concerns regarding *in vivo* use due to cytotoxicity, short half-lives, poor solubility, and instability^2^. On the other hand, polymers have an inherently complex structure and low efficiency as transfection reagents^3^.

Polyamidoamine (PAMAM) dendrimers represent a class of hyperbranched polymers that are versatile vehicle candidates in nanomedicine, especially in the fields of diagnosis and cancer therapy^4^. PAMAM dendrimers consist of a central core with multiple emerging branches, which over classical polymers, the functions of which cannot be precisely controlled. Dendrimer terminal groups are also critical in binding several types of drugs^5^,^6^ and in influencing dendrimer interactions with the cell membrane and cytoplasmic proteins^7^.

Dendrimers present the following advantages compared to traditional transport molecules^8^: 1) multipurpose control over surface groups; 2) excellent cell uptake, which provides high drug bioavailability; 3) monodispersity and manageable size, which facilitates biomedical applications; 4) globular architecture that resembles a protein, which enables application without an immunoreaction^9^; and 5) high nucleic acid affinity and the ability to release drugs, which prevents complications during cancer therapy^10^.

Due to a positive charge at physiological pH, PAMAM-NH_2_ dendrimers are efficient nucleic carriers^11^,^12^. Terminal groups theorised to increase the gene carrying abilities of dendrimers include amino acids^13^,^14^, cell-penetrating peptides such as GALA^11^, and polyethylene glycol groups on dendrimer surfaces^15^, among others.

A novel type of PAMAM-based carrier, termed amphiphilic dendrimers (ADs), was described by Yu *et al*.^16^, whose goal was to develop an ideal vector that displayed cell membrane lipid affinity and the ability to bind to the DNA of cationic dendrimers. These lipid/dendrimer hybrids, which are able to self-assemble in solution, are comprised by a hydrophilic, low-generation dendron and a long, hydrophobic tail. Moreover, the self-assembly capacity of ADs could result in a highly multivalent structure similar to that of high-generation dendrimers, but at a lower cost, since an energetically demanding purification process is not needed^17^.

Yu *et al*.^16^ also demonstrated that these new ADs can deliver small-interfering RNA (siRNA) and produce a notable gene-silencing effect. Three generation 2 (G2) ADs were synthesized with different alkyl tail lengths that contained either 18 (18C), 15 (15C), or 13 (13C) methylene groups. Whereas 18C ADs were reported as efficient siRNA vectors, showing significant gene silencing, 15C and 13C failed as transfection systems. Furthermore, G1 and G0 ADs bearing 18C tails also failed to elicit efficient transfection. Currently, there is no clear explanation for the molecular basis of these phenomena.

Tschiche *et al*.^18^ more recently synthesized a set of polyglycerol-based, low-generation amphiphiles (G1 and G2) that are surface functionalized with DAPMA (N,N-di-(3-aminopropyl)-N-(methyl)amine) moieties. These new ADs are similar in size and molecular weight, to those described by Yu *et al*.^16^, the radii of which fluctuate between 1.5 and 2.5 nm. Amphiphilic substances, such as nanocarriers, can form micellar, liposomal, or lamellar structures. Micelles are formed when the hydrophobic portions generate a core to avoid water while the hydrophilic sections orient towards water. Micelles have a variety of applications^19^, and polymeric micelles, such as block copolymers, have been extensively studied as a drug-delivery method that encapsulates drugs with poor solubility. The hydrophobic portion of amphiphilic molecules plays an important role in DNA condensation^20^,^21^,^22^. Related to this, experiments using optical tweezers show different DNA binding modes depending on the hydrophobic chain length of surfactant molecules. Short-chain molecules can lie on a nucleic acid surface while long-tail chain molecules tend to point away from the surface^20^.

Notably, several computational studies have already been performed that provide insight into the self-assembly of ADs. One of the most common techniques, dissipative particle dynamics, was used to study stochastic AD assembly in presence of DNA^17^,^23^. In early mesoscale methods, such as dissipative particle dynamics, multiple molecules are constrained into beads^24^. Later, classical CG molecular simulations were implemented to simplify the exploration of structural and dynamic properties in large systems^24^. The results of CG molecular simulations not only highlight the usefulness of these simulations in overcoming the time scales of conventional molecular dynamics (MD), but also reveal important information regarding lipid self-assembly^25^,^26^,^27^. Finally, the MARTINI force field, which can be applied to CG molecular simulations, is a maximally transferable force field designed to represent information on the free energy of each molecular component^24^.

The present study modelled the AD systems introduced by Yu *et al*.^16^ to deepen understandings on micelle formation and siRNA binding, as well as to explain the successful results of G2-18C ADs as gene vectors. To this end, our group began systematic research on the structural and energetic properties of ADs through coarse-grained (CG) molecular simulations. The computational approach proposed in this work was applied with the aims of molecularly comprehending how ADs of different generations and alkyl chain lengths are able to self-assemble and how the size and shape of the assembled structures impact siRNA binding.

## Experimental Section

### Coarse-Grained Modelling of Amphiphilic Dendrimers

The MARTINI CG model, which is widely used to simulate lipids, proteins, and carbohydrates, employs four interaction “bead” types: polar, apolar, nonpolar, and charged. These types can be sub-classified into 18 types of interactions according to hydrogen-bonding capabilities and polarity^28^. This model was extended to dendrimers, specifically PAMAM dendrimers^29^,^30^, and is able to distinguish dendrimer nodes and branches. The parameters of this model are available on the MARTINI website (md.chem.rug.nl/cgmartini/images/parameters/ITP/martini_v2.0_polymers.itp) and were adapted in the present study for use with the dendritic portion of ADs.

The ADs originally proposed by Yu *et al*.^16^ are composed of G2 dendrons with eight amine-terminal groups and three different alkyl chains (18C, 15C, or 13C). To assign a CG model for hydrophobic chains, the present study considered the lipid force field developed by Marrink *et al*.^26^ In the CG model, a single bead replaces four methylene units. Therefore, the present study used 5 beads for an 18 carbon-tail structure, 4 beads for a 15 carbon-tail structure, and 3 beads for a 13 carbon-tail structure. One hydrophobic bead was assigned to represent the ring between the dendron and the tail.

**Figure 1:**
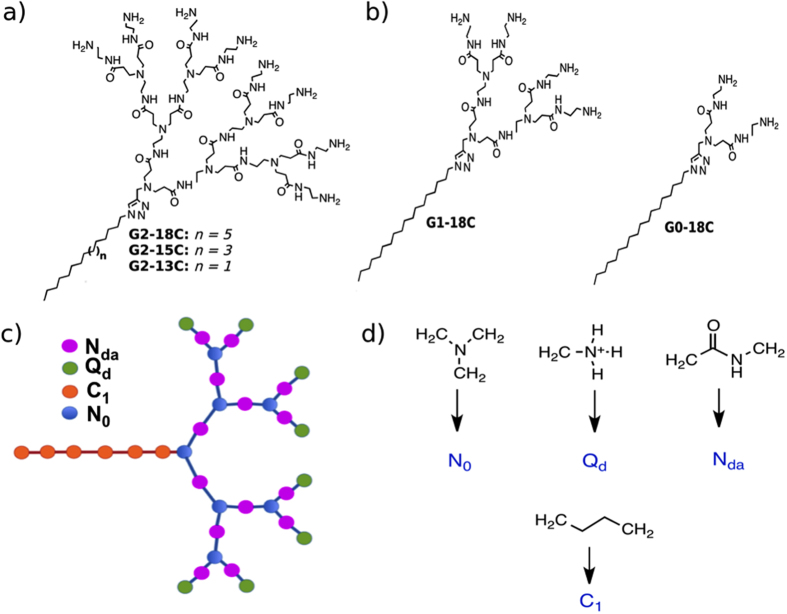
(**a**) As described by Yu *et al*.^16^, full-atom chemical structure of AD, composed of a G2 dendron section and an aliphatic tail, having 18 carbons (n = 5), 15 carbons (n = 3) and 13 carbons (n = 1). (**b**) Full-atom chemical structure of AD composed of a G1 or G0 dendron section and an 18 carbons aliphatic tail (**c**) Structure of 18C tail-length AD using CG beads. (**d**) Mapping of dendron chemical groups, as described by Lee and Larson^29^,^30^. and CG C_1_ tail beads as described by Marrink *et al*.^26^, in the CG lipid force field.

In previous research, Yu *et al*.^16^ also synthesized G1 and G0 dendrimers with four and two amine-terminal groups, respectively, and 18-carbon tails. These structures were also considered in the present molecular study to address the influence of AD generation in siRNA binding. The pH titration profile of Yu *et al*.^16^ determined that terminal primary amines are protonated at a physiological pH; therefore, all of the dendrimer terminal amines assessed in the present study were considered protonated. The structure and bead mapping of ADs are shown in Fig. 1.

Full atomistic simulations of single ADs in explicit water were transformed to CG resolution using the mapping technique described in Fig. 1. Equilibrium values and force constants for bonded interactions were measured using mapped-to-CG atomistic simulations and then represented using histograms, as recommended by Martini *et al*.^24^,^31^ The radii of gyration were obtained from the CG models and contrasted with full atom models for confirmation. Further details of full-atom simulations are described in Supporting Information.

**Figure 2:**
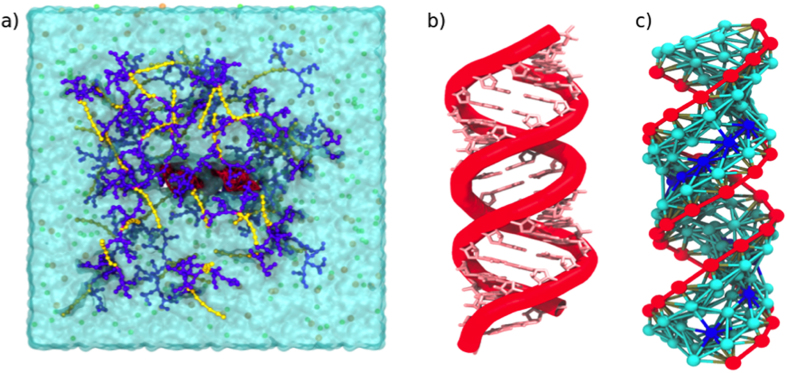
(**a**) Snapshot of the initial CG system composed of 175 ADs and a single molecule of siRNA (red), placed at random into a water box with counterions. Amphiphilic dendrimers are represented by a headgroup (purple) and a hydrophobic tail (yellow). (**b**) Fragment of the full-atom structure of a double-stranded RNA (PDB ID: 4E48). (**c**) CG model of the 20 base-pair siRNA considered in the present study.

A single siRNA molecule and 175 ADs were placed at random into a 37.6 nm^3^ × 37.4 nm^3^ × 37 nm^3^ non-polarized Martini water box, representing a molar concentration of 13 mM of ADs. (Fig. 2a). Five systems (G2-18C, G2-15C, G2-13C, G1-18C, and G0-18C) were considered. NaCl (150 mM) was also added to each box, with each ion represented by a single CG bead.

The simulations were carried out using the GROMACS package (version 5.0.3)^32^. The method of steepest descent was employed for energy minimization using a force tolerance of 10 kJ mol^−1^ nm^−1^. Then, MD simulations in the isobaric-isothermal ensemble were performed under periodic boundary conditions with a constant temperature of 310 K, measured using the velocity rescaling thermostat (modified Berendsen)^33^, and at 1 bar of pressure, controlled using the Parrinello-Rahman scheme. Short-range electrostatics and Lennard-Jones potentials were shifted from 0.0 and 0.9 nm, respectively, to the cut-off distance (1.2 nm) using the standard shift function in GROMACS^34^. Long-range electrostatics were calculated using Particle Mesh Ewald summation^35^. Verlet algorithm and an integration time step of 30 fs were considered. The total simulation length for each system and their replicas was 5 μs.

### Coarse-Grained Modelling of siRNA

In Yu *et al*.^16^, a 20 base-pair siRNA fragment was investigated that targeted heat shock protein 27 in human, castration-resistant, prostate cancer PC-3 cells. The present study built a full-atom siRNA model (sense, 5′-GCU – GCA - AAA – UCC – GAU - GAGAC dTdT-3′; antisense, 5′-GUC -UCA – UCG – GAU – UUU – GCA – GC - dTdT-3′) based on a double-stranded RNA crystal structure deposited in the Protein Data Bank (www.pdb.org) (PDB ID: 4E48). The CG model was adjusted to the full-atom structure (Fig. 2b).

Khalid and Corsi *et al*.^36^,^37^ developed a CG DNA model based on MARTINI CG beads, using one bead for phosphate groups, two for deoxyribose sugars, and two or three beads depending on the base types. Furthermore, bonding interactions of DNA were treated using an elastic network approach, where all particles separated by distances up to 0.7 nm were restrained with a harmonic potential of 1500 kJ mol^−1^ nm^2^. Particles separated by a higher distance only interacted under non-bonding conditions. The equilibrium bond-lengths were taken from a B-form of DNA and validated by Khalid and Corsi *et al*.^36^,^37^ using several full-atom MD simulations in water and counterions.

The Corsi’s approach was applied in the current study to generate a siRNA force field with similar equilibrium constants. However, the distance between atoms was taken from the aforementioned crystal structure. Also based on Khalid and Corsi *et al*.^36^,^37^, the same bead types were employed for phosphate groups, as well as for guanine, cytosine, and adenine. Two *Na* particles were used for uridine. A polar bead (*P1*) was assigned to the ribose sugar groups. The types of particles used to build the siRNA model are summarized in Table 1.

## Results and Discussion

### Dynamics of micelle formation

**Figure 3:**
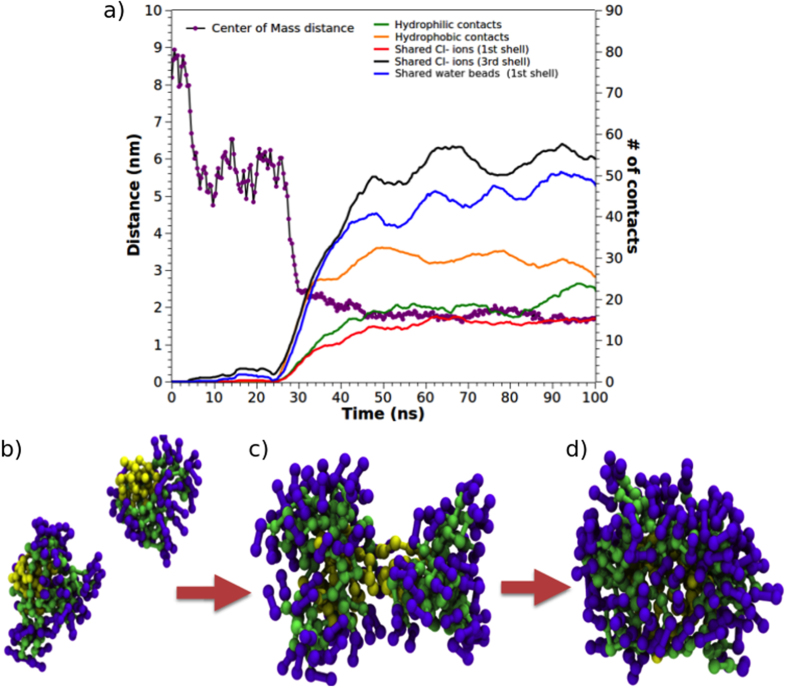
Two micelles from the G2-18C system were considered for analysis. The COM distance was used as an indicator for the micelles. (**a**) Different types of contacts were inspected to determine the nature of fusion. Snapshots of the micelle formation process: (**b**) long-distance contact (0–35 ns), (**c**) short-distance contact (35–55 ns), and (**d**) formation of the final micelle.

To characterize the events driving self-assembly of this class of amphiphilic dendrimers, the fusion of two aggregates from the G2-18C system was considered as a case-of-study to verify if these systems are acting as typical ionic surfactants (Fig. 3a). More than 10 of these events were detected in the simulations of each system. To identify the recurrent events observed in self-assembly, a short trajectory fragment was analysed. Specifically, two micelles separated by a centre-of-mass (COM) distance of ≈9 nm and with hydrophobic portions (tails) exposed to the solvent were assessed (Fig. 3b). The first contact between these initial micelles began through anionic counterions that generated a long-distance contact network (0 to 30 ns). Then, the COM distance between the initial micelles plateaued at ≈6 nm for 20 ns (35 to 55 ns). In this step, initial long-distance contacts were established through the generation of an interaction network promoted mainly by counterions that were part of the second or third ion solvation shell of the initial micelles. The micelles remained at this distance for a short period, allowing the tails of each micelle to reorient towards one another. During this short period, there was an initial hydrophobic collapse among the tails of the initial micelles, as represented by increased short-distance interactions among the hydrophobic groups and by the loss of entropy of water in direct contact with the hydrophobic tails. As this relaxation period occurred, the hydrophilic contacts and chloride ions shared between the initial micelles increased (Fig. 3c). The distance between the COM of the micelles then decreased (50 to 55 ns) until a second plateau was reached at 2 nm. This generated a short distance contact fostered by the sharing of chloride ions and was stabilized through the interaction with the positively charged groups of AD heads.

As expected, the number of chloride ions shared by the solvation shells of both micelles was key to the catalysis of the self-assembly reaction, as it was widely described for ionic surfactants^38^. In this event, water also played an important role by creating bridges between chloride ions. The amount of chloride ions shared by the initial micelles plateaued when the micelles appeared totally merged. At the time of fusion, the shared solvation shells were represented by a peak of shared water molecules between the hydrophobic portions of both micelles. This peak decreased as the micelles completely fused.

Finally, the amount of shared water beads increased during the first period of the second plateau (55 to 75 ns). This event was followed by decreased sharing due to increased contact density between the ADs that generated a final micelle (Fig. 3d).

### Characterization of amphiphilic dendrimer self-assembly

As mentioned above, five MD simulations lasting 5 μs each were carried out in the presence of a single siRNA molecule to monitor the self-assembly of the different ADs into micelles. The aim of these simulations was to gain insight on the influence of AD generation and alkyl chain length on interactions with siRNA. Morphological characterizations of the aggregates formed in systems containing siRNA are summarized in Table 2. Additionally, Supporting Fig. S1 shows the distribution of micelle radii in each system.

In the three systems with the same generation dendron (G2) but different tail lengths (18C, 15C, and 13C), the micelle radii, obtained via the radii of gyration, were between 1.8 and 2.4 nm, with an average of 2.1 nm.

Proposed by Israelachvili^39^,^40^, the molecular packing parameter represents how the size and shape of an aggregate at equilibrium can be predicted through molecular packing considerations and general thermodynamic principles. The molecular packing parameter *P* of a given amphiphile is defined by Equation 1 as:


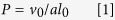


where *v*_0_ is the volume of the hydrophobic chain; *a* is the surface area of the hydrophobic core of the aggregate, expressed per aggregate molecule (i.e. the area per molecule); and *l*_0_ is the critical length of the hydrophobic tail, as shown in Supporting Fig. S2.

According to Israelachvili, a packing parameter ≤0.33 represents a spherical micelle. A value between 0.33 and 0.50 represents a cylindrical structure, and a value ≈0.50 represents a bilayer. These rules predict that a large headgroup, or high a_e_, tends to form spherical micelles, while surfactants with a small headgroup, or low a_e_, tend to form cylindrical micelles or lamellae.

Considering a generic micelle with a core radius *R*, made up of *N* molecules, then the volume *V* and the surface area *A* of the core can be determined by Equations 2 and 3:


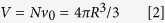



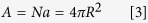


From simple geometrical relationships^39^, the core radius *R* can be determined by Equation 4:


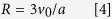


Accordingly, the packing parameter was calculated for each micelle in the five systems. This data showed that all aggregates formed from G2-18C and G2-15C ADs were spherical, monodisperse micelles, whereas at least one aggregate from the G2-13C, G1-18C, and G0-18C systems was not spherical, tending to adopt a “rod” shape. These “rod” micelles were also able to bind siRNA, but the number of charged amine groups in contact with the nucleic acids was differently distributed. While this will be discussed in depth in the section entitled “Distribution of Ions and Water beads around siRNA”, the transition from a sphere to rod is behavior described for other amphiphilic systems^41^.

The mean surface area of the micelles binding siRNA tended to be higher (57 nm^2^) for the G2-18C and G2-15C systems than for G2-13C (49 nm^2^). This value was related to the core radius, which was larger for 18C and 15C ADs due not only to the greater number of C atoms than 13C ADs, but also due to the degree of curvature, or packing, of the tails inside the core. Notably, dendrimersomes can form as a result of the self-assembly of ADs bearing two alkyl chains^42^. The present study determined that the most stable dendrimersomes were those that formed the thinnest bilayers as a result of the interdigitation of the alkyl groups. Interdigitation could promote a more dense hydrophobic cluster with less interstitial space, which would contribute to better packing and a more stable core. Considering these details, the mean tail length and core radius of every micelle were estimated. The ratio between tail length and core radius (I_d_) can be calculated as a measurement of interdigitation degree, where I_d_ = 1 represents a micelle with tails that are not interdigitated. The higher the value of I_d_, the higher the interdigitation degree. Therefore, micelles composed of 18C ADs had a mean degree of interdigitation ≈1.5; G2-15C ADs had ≈1.3, and G2-13C had ≈1.1. To compare the degree of core packing in micelles composed of the same number of dendrimers, the radial distribution function was calculated for the tails of two 19 AD micelles of G2-18C and G2-13C (Fig. S3, Supporting Information). Under the radial distribution function, the volume occupied by the hydrophobic tails is proportional to the area under the RDF. Based on this, the area for the G2-18C tails was calculated to be ≈1.2-fold greater than for the G2-13C tails. This evidence supports the idea that 18C tails would promote a higher degree of packing, resulting in more stable micelles.

In systems with 18C tails but different dendron heads, G1-18C formed more similarly sized micelles than the G2-18C ADs (mean radius ≈ 2.1), but the interdigitation degree of the tails from the G1-18C system (≈1.1) was closer to the G2-13C system. This suggests that less stable micelles were formed in the G1-18C system. As further discussed later, G0-18C system ADs were different because a large cylindrical micelle was formed along with two other much smaller and poorly structured micelles. In a hypothetical system with a higher concentration of ADs, more cylindrical micelles would form, thus preventing the formation of smaller micelles. In G0-18C system, the interdigitation parameter only considers the cylindrical micelle and the minimum radius. Therefore, a tail length of 1.7 nm and minimum cylinder radius of 1.5 nm would have an interdigitation parameter of ≈1.1. In summary, micelles originating from 18C ADs appeared more structured. Based on core volume, G0-18C micelles could also be a promisory hydrophobic drug carrier due to a propensity to encapsulate a high quantity of drug molecules in the hydrophobic core, thereby increasing solubility inside cells. Nevertheless, the G0-18C system offers few charged or polar groups that could generate electrostatic and H-bond networks with other substrates.

To corroborate that ADs are able to form micelles even in the absence of siRNA, three MD simulations were conducted. G2-18C (Fig. S4, Supporting Information), G2-15C, and G2-13C ADs were observed under the same salt concentration and for 10 μs, 5 μs, and 5 μs respectively. The number of ADs for each aggregate, as well as the number of aggregates, were inspected each microsecond (Figs S5, S6, and S7, respectively, Supporting Information). The size of the aggregates are summarized in Table S1, Supporting Information. The ADs formed similarly sized micelles (≈2 nm radius) in systems with and without siRNA. Furthermore, the solvent accessible surface area (SASA) of the hydrophobic portion, as a percentage of the total SASA of each micelle with different AD quantities, was calculated to study the relationship between solvent accessibility and the size and number of aggregates (Fig. S8, Supporting Information). These calculations determined that the SASA of hydrophobic portions decayed until plateauing at 2% solvent accesibility, while micelle diameter increased over 3.2 nm. This indicates that in micelles with a diameter ≈3.2 nm, the hydrophobic portion is inaccessible by the solvent, preventing collapses with other micelles and stabilizing micelle structures.

### Dynamics of siRNA interactions with self-assembled structures

Rapid micelle formation in the presence of siRNA was detected in the CG simulations of ADs. To evaluate the dynamics of micelles binding to siRNA, the COM distance was estimated between each micelle and siRNA (Fig. S9, Supporting Information). In the first microsecond of the simulation, micelles were not completely formed and presented a COM distance equal to the mean distance of several ADs not yet assembled into an aggregate.

**Figure 4:**
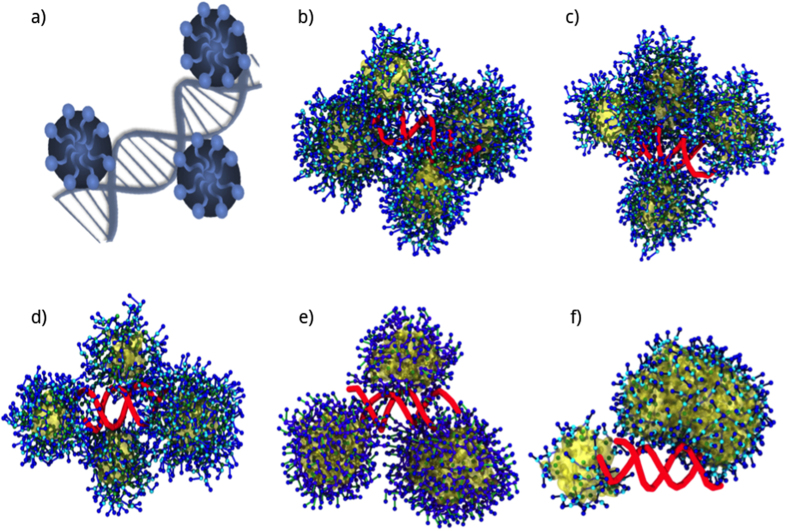
(**a**) Representation of DNA condensation by cationic surfactants forming spherical micelles, as reported using electron microscopy^46^. Last frame (5 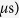 snapshot of each MD trajectory, showing the formation of complexes between siRNA and AD micelles for the (**b**) G2-18C, (**c**) G2-15C, (**d**) G2-13C, (**e**) G1-18C, and (**f**) G0-18C systems.

Significantly, in the three systems with different alkyl chain lengths and G2 dendrons, four micelles bound to siRNA based on the distance to nucleic acid (≈2–5 nm). Therefore, four micelles of each system remained at a stable COM distance from siRNA during the simulations, forming a large nanoparticle. In the case of G2-13C, the shortest tail system, a fifth micelle remained near the cluster of four micelles. The small size of the four G2-13C micelles allowed several siRNA phosphates to remain free, which could have promoted the approach of the fifth micelle (Fig. 4).

For ADs from different generations but with the same chain lengths (18C), only three micelles (G1) and two micelles (G0) were able to bind to siRNA. This reduced number of micelles could expose more siRNA to the environment. The G0 and G1 micelles were larger than the G2 micelles. However, the increased size produced more rigid G0 and G1 aggregates than those of G2. Moreover, G2 offered increased surface contact with siRNA when compared to larger aggregates. Significantly, the four micelles in the G2 systems more efficiently covered the total surface of the nucleic acid.

In a recent study performed by Kasyanenko and coworkers^43^, the DNA binding to cis- and trans-isomers of azobenzene containing surfactant was explored through experimental and computational studies. Classical molecular dynamics simulations evidenced the spontaneous formation of micelles of similar shape for both isomers. As in the present study, stable adsorption of surfactants assembles was favored over single structures throughout the simulation. Moreover, the same beads-on-thread organization was registered for the trans-isomer, which fitted the minor groove of the DNA helix.

**Figure 5:**
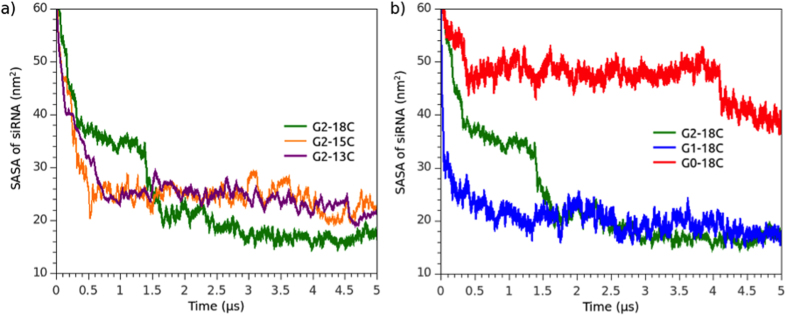
SASA of siRNA in the presence of dendrimers (**a**) G2-18C, G2-15C, and G2-13C; and (**b**) G2-18C, G1-18C, and G0-18C. Towards the end of the simulation, the siRNA fragment was almost completely covered by the dendrimers in each system.

To evaluate the coverage of siRNA phosphate groups in the presence of micelles, the SASA was calculated to measure the interactivity of micelle ADs with siRNA. High siRNA SASA values represented micelles that provided weaker environmental protection. SASA values were obtained over the total simulation time of each system (Fig. 5). Results of SASA from an additional replica for each system are depicted in Fig. S10, Supporting Information. In all of the analyzed cases, siRNA SASA decreased until reaching a plateau. The G2-18C system had better siRNA coverage than the G2-15C and G2-13C systems. The data obtained by contrasting the siRNA SASA and its distance from micelles (COM data) suggest that G2-15C and G2-13C dendrimers are not able to form micelles that efficiently bind to siRNA due to shorter alkyl chains that promote the formation of less stable micelles. For G2-13C ADs, the chemical equilibrium that drove micelle stability would promote a higher AD interchange between micelles. In general terms, there was an equilibrium between dendron-siRNA and tail-tail interactions in stable micelles. Among the short-tailed dendrimers, such as G2-13C, dendrimer-siRNA interactions could be stronger than tail-tail interactions, which would result in lost packaging density and stability.

G2-13C micelles that effectively bound to siRNA had, on average, a smaller radius (≈1.9 nm) than the micelles that bound to siRNA in the G2-15C and G2-18C systems (≈2.1 nm) (Table 2). This suggests that the siRNA SASA covered by micelles in te G2-13C system was slightly less than that of G2-15C and G2-18C micelles. Moreover, the sterical hindrance imposed by small 13C micelles could be an obstacle for additional micelle binds with siRNA.

In the G0-18C and G1-18C systems, G0 showed the highest SASA values while G1 showed a profile similar to the G2-18C system. This indicates that the structure and number of charged groups of a G0 head do not form stable micelles, and, therefore, are not able to bind efficiently to siRNA. The formation of the large “rod” seen in G0 could induce a steric repulsion that prevents the aggregation of more micelles instead of forming a protective layer. While G1 can efficiently bind to siRNA, fewer micelles were formed during the G0-18C/siRNA complex simulation, as indicated by the respective COM profile.

**Figure 6:**
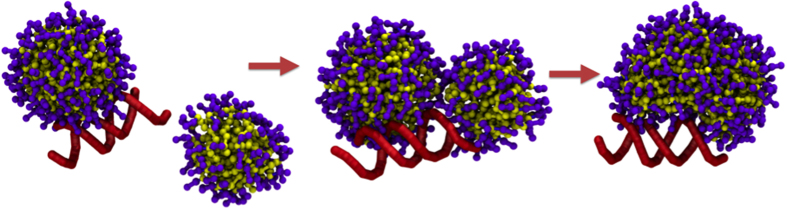
Snapshots of the formation of a G0-18C rod. The siRNA backbone is depicted in red.

In the simulations and their respective replicas, several micelles were formed in the G2-18C system, but only four remained close to siRNA, whereas the other ones tend to get closer, but they do not interact with siRNA nor merge with the four micelles. In contrast, almost all G0-18C dendrimers merge to one of the aggregates that bind siRNA, forming a big rod, bigger than G2-18C micelles, which was observed in both original and replica simulations. Snapshots of the last frame of both systems and their respective replicas are depicted in Fig. S11, Supporting Information. As described by Jones *et al*.^23^, micelle surface charge density σ_m_ = eN_agg_/S_m_ can be thought of as a measure of micelle charge as a function of surface area S_m_. When considering as an example, two G0-18C micelles before rod formation, equivalent in size to the G2-18C micelles, one with N_agg_ = 70 and the other with 40 ADs (two amines per AD, e = + 2) and with respective micellar surface areas S_m_ equal to 50.2 nm^2^ and 36.3 nm^2^, the σ_m_ for these systems were estimated as 2.6 and 2.1 e/nm^2^. Applying this same calculation to two G2 micelles of N_agg_ = 13 and 19 (Table 2), the σ_m_ were estimated as 3.4 and 3.1 e/nm^2^. Thus, these G2 micelles had a higher number of charges per surface area than the G0 micelles of similar size. These higher charge densities helped to stabilize a stronger network of counterions around the micelles and contributed to the electrostatic repulsion among micelles, preventing their merge. In contrast, G0-micelles form a bigger aggregate (Fig. 6), differently to the behaviour presented in the other G2 and G1 systems. As mentioned above, the formation of rod is an expected behaviour in amphiphilic molecules of small polar head and long hydrophobic tails. Yamamoto *et al*.^44^ described long-tailed, small-tailed and double-tailed amphiphiles using a coarse approach, observing sphere-to-rod events. Later, Velinova *et al*.^41^ have described these events in a PEG-alkyl amphiphile. In this system, small PEG fragment acts as a “worm”, which is not forming a compact and big head as G2-18C, but a small-headed amphiphile such as G0-18C dendrimers.

### Distribution of ions and water beads around siRNA

**Figure 7:**
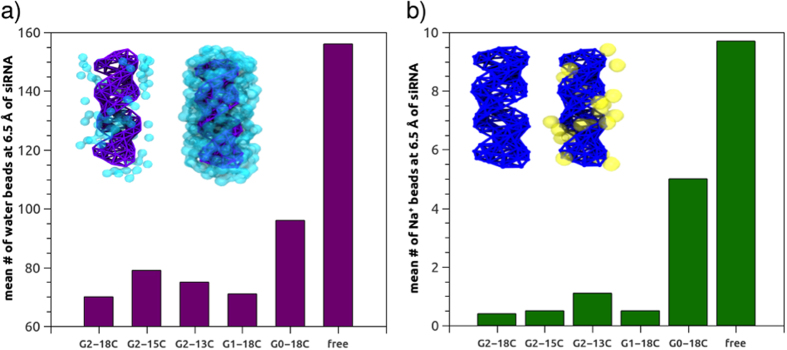
(**a**) Mean number of water beads and (**b**) sodium beads 0.65 nm from the COM of siRNA. Number of water and sodium beads for free siRNA in the solution is provided for reference. Inset of (**a**,**b**): Volume maps of siRNA locations most commonly occupied by water and sodium beads, in contact with micelles (left) and free in the solution (right). Values for free siRNA were taken from a simulation containing 150 mM NaCl, as in the AD/siRNA systems.

To elucidate how water and counterions are displaced from the siRNA solvation shell in the presence of different ADs, the mean number of water beads and counterion beads in contact with siRNA (at 0.65 nm; Fig. 7) was calculated over the last μs (4–5 μs) of MD simulations, after the formation of complexes between siRNA and dendrimers. It must be noted that one MARTINI water bead represents four water molecules. Since siRNA alone in a solution, as a polyanion, is compacted and neutralized by sodium ions, its displacement from the siRNA solvation shell upon complexation with micelles was analysed. Values observed in a molecular simulation of free siRNA (150 mM NaCl) were recorded. From these simulations, the G2-18C dendrimers appeared to act as protective structures for the siRNA since the distribution of water beads around the nucleic acid was the lowest among the observed systems. Consequently, the number of water beads around siRNA in complexes with these dendrimers was lower than in the case of free siRNA in the solution (Fig. 7a). Water beads were still present in the vicinity of siRNA in the micelles of the G2-15C and G1-18C systems, but the sodium beads in these systems appeared completely displaced (mean number <1; Fig. 7b).

**Figure 8:**
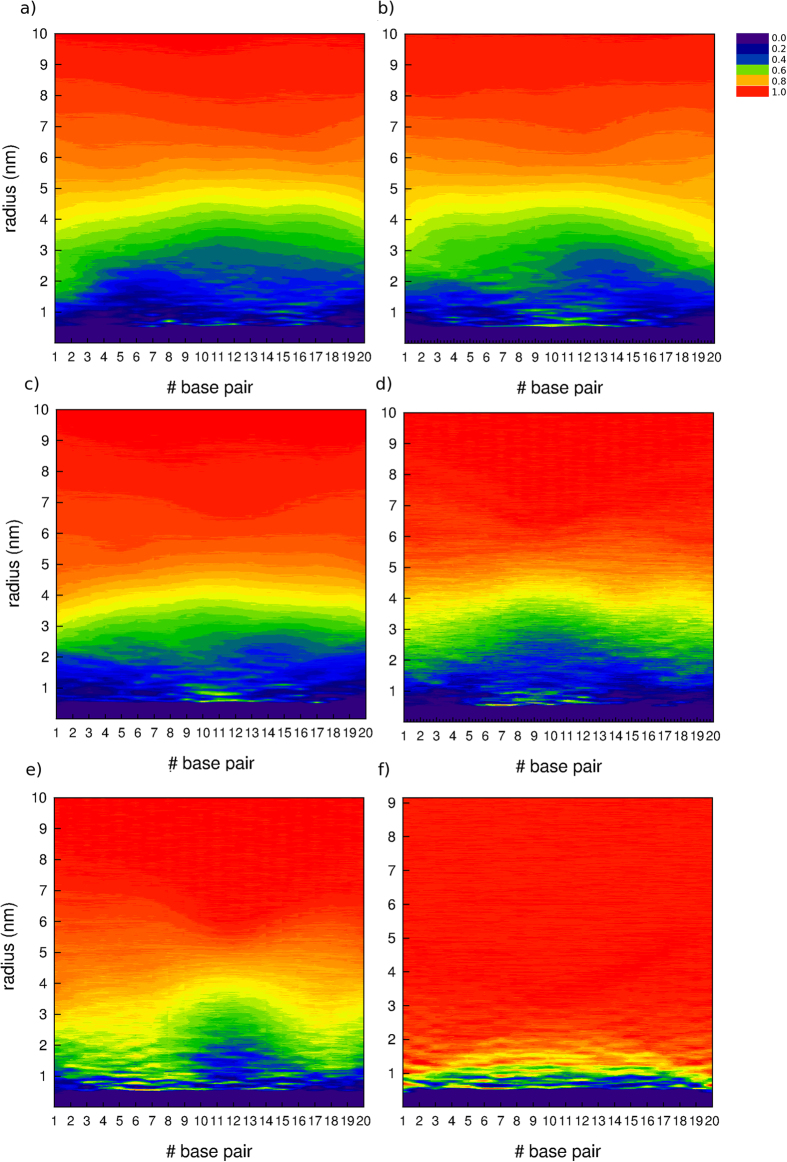
Radial distribution function (RDF) of water as a function of the distance from each siRNA base-pair centre-of-mass (COM), for each system (**a**) G2-18C, (**b**) G2-15C, (**c**) G2-13C, (**d**) G1-18C, (**e**) G0-18C. Data of the distribution of water in a system containing only siRNA have been added as a reference (**f**). Each base pair is represented in the x-axis, radius (nm) in the y-axis and surface in colours (z) represents the RDF. A small fringe along the x axis, where y < 0.5 nm (purple), represents the zone where siRNA atoms are present.

Additionally, radial distribution functions of water (Fig. 8) and sodium counterions (Fig. S12, Supporting Information) as a function of the COM of each siRNA base pair were calculated from the last microsecond for each system, obtaining a 2D view of density around siRNA. Control densities of water and ions were also obtained from a MD simulation of coarse-grained siRNA free in solution. From these data, it can be seen that densities of water and ions drop to zero near the zones of siRNA that are more protected by the aggregates, denoted in the plots with blue tonalities. Therefore, the plots show how progressively blue areas are reduced compared to the G2-18C system, while decreases the generation of the ADs and alkyl-tail length. Specially, in terms of ion density, G2-18C appears as the system with lower density of counterions in the first solvation shells of siRNA, showing a quite uniform profile along the base pairs, which suggests a better performance of these ADs in protecting siRNA from the environment. Several consecutive dots, representing counterions density values close to 1 in the vicinity of siRNA in G2-15C and G2-13C systems, can be explained by a single counterion that was trapped in the micelle-siRNA interface. Due to the radial calculation of the density, it is clear that these dots appear along several base pairs. Density profiles of water and specially, counterions also appear delineating the shape of siRNA. RDF data revealed once again that G0-18C dendrimers were not able to efficiently displace the water and ions beads from the solvation shell of siRNA. Instead, just a few water beads remained close to the COM of siRNA base pairs #10 to #15, judging by the low densities of the water RDF in those zones, while the rest of siRNA appears more deprotected, having similar water and ion densities than in siRNA free in solution system. Moreover, this evidence reinforces the idea that G0-18C aggregates are not efficiently protecting siRNA from the environment, as SASA data previously shown.

### Distribution of amphiphilic dendrimer contacts with siRNA

**Figure 9:**
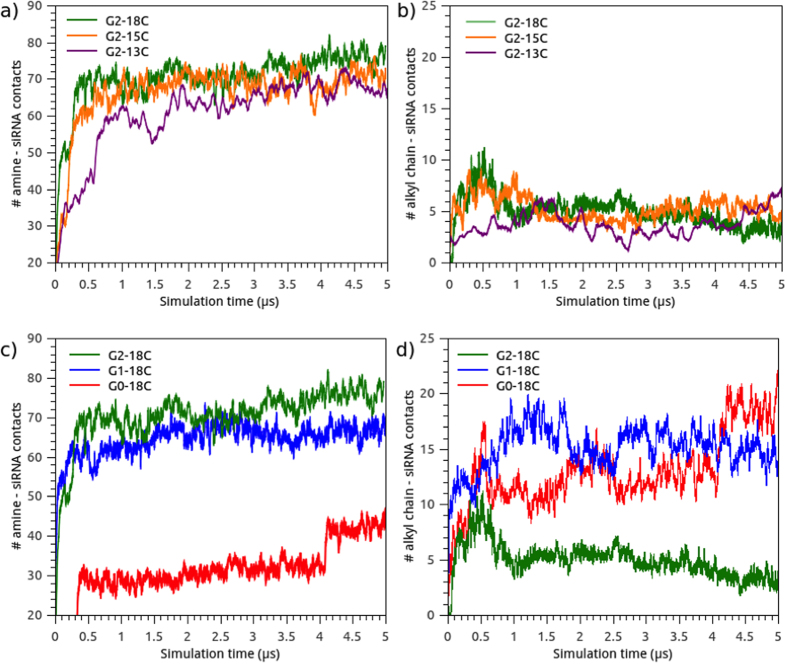
Time evolution for the number of contacts between amine groups of dendrimers and siRNA at 0.75 nm and alkyl chains and siRNA at 0.75 nm. (**a**) and (**b**) represent data for G2-18C, G2-15C, G2-13C and (**c**) and (**d**) for G2-18C, G1-18C, G0-18C.

Electrostatic interaction is the most important binding force involved in the formation of a nucleic acid-dendrimer complex^45^. Since amphiphilic dendrimers are composed of amine-terminated PAMAM dendrimers, the number of contacts between siRNA and amine groups within a 0.75 nm radius was calculated as a measure of complex stability (Fig. 9a,c). As expected, the interaction of the amine groups with siRNA was inversely correlated with the degree of siRNA exposure to the solvent (i.e. SASA). Amine groups allowed the formation of a complex, with constant interaction from 2 μs to the end of the simulation for every system. Considering this parameter, the only system that behaved differently was G0-18C, where, in the last fragment of the simulation, the siRNA appeared to be covered by a small aggregate, in addition to a large rod.

To verify the importance of alkyl chain length in siRNA interactions, the number of contacts between siRNA and the tail groups were also calculated within a radius of 0.75 nm (Fig. 9b,d). Interestingly, during the first 0.5 μs, the interaction between siRNA and hydrophobic tails peaked in regards to the number of contacts in systems with 18C and 15C tails. In the first stage, the hydrophobic tails aided in triggering AD-siRNA complex formations until the electrostatic contributions gained more relevance (≈1 μs) and the micelles stabilized. At this point, hydrophobic collapses decreased, and the electrostatic interactions between amines and siRNA remained stable for the remaining simulation time. The interaction of hydrophobic groups with nitrogenized bases of siRNA was more pronounced in the G0 and G1 systems. In these systems, the hydrophobic groups are closer to siRNA due to small headgroups. Results of number of contacts from an additional replica for each system are depicted in Fig. S13, Supporting Information.

### Amine-siRNA phosphate contacts as a measure of siRNA affinity

The primary factor involved in nucleic acid condensation by dendrimeric nanostructures is the ratio between positive dendron headgroups and phosphates of DNA/RNA (N/P ratio), which is highly dependent on the size and shape of the aggregates. Previous evidence demonstrated that the rigid structure of DNA could affect the organization of micelles and induce the elongation of micelles into “rods.” This elongation results in an environmental exposure of DNA portions, which is an unwanted outcome since, in gene delivery applications, nucleic acid must remain protected from the environment^46^.

**Figure 10:**
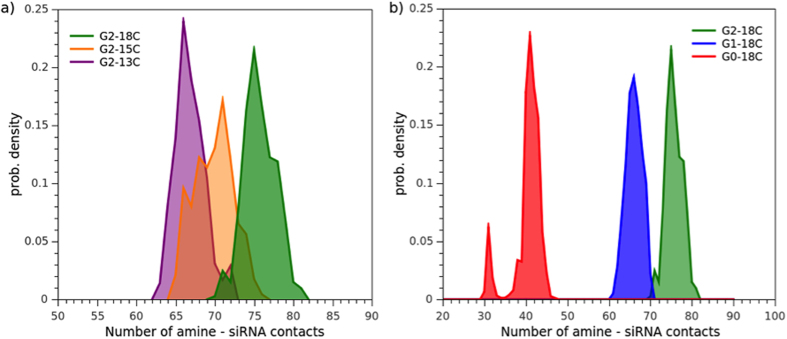
Histogram of the number of contacts between amine-terminal groups of the dendrimers and phosphate groups of siRNA, for (**a**) G2-18C, G2-15C, G2-13C and (**b**) G2-18C, G1-18C, G0-18C. Density was normalized considering the total values recorded for the last microsecond of simulation.

For this reason, the total number of dendron amine groups interacting with siRNA, within a cutoff of 0.75 nm, was determined for each frame over the last microsecond of MD simulation, once all micelles were completely structured (Fig. 10). Results for the respective replicas are depicted in Fig. S14, Supporting Information. The mean number of amine groups per micelle that bound to siRNA is detailed in Table 3.

The mean quantity of amines that bound to siRNA for the G2-18C system was ≈75, greater than the G2-15C and G2-13C systems (≈70 and ≈67 amines, respectively). This indicated a lower binding enthalpy, greater stability, and, thus, superior dendrimer affinity for siRNA in the G2-18C system. The size, shape, and formation of the aggregates were influenced by the alkyl chain length and affected siRNA interaction, despite the three dendrimers having the same number of amine-terminal groups. The longer alkyl chains promoted bigger micelles with lower curvature degrees that allowed for more contacts with siRNA, unlike shorter alkyl chain dendrimers.

By taking into account the number of G2-18C dendrimers that form the micelles required to completely bind to siRNA (i.e. 13, 19, 24, and 26 AD micelles), the N/P ratio can be calculated as 82/40 = 2:1, which is a higher estimator than if considered the number of amines that actually are binding siRNA. These results can be related with a previous ethidium bromide exclusion assays that found these dendrimers to completely retard the migration of siRNA in a gel at N/P ratios above 1^16^. It must be noted in gel retardation experiments, it is not possible to detect how much AD is bound to siRNA and how much AD is free in solution, thus this N/P ratio is probably a higher estimate of real N/P of the complex, similarly than the estimations from MD simulations.

The differences were more pronounced when comparing systems with different dendron generations. The sizeable micelle formed by G0-18C dendrons should promote more contacts with siRNA, even if the number of amines per dendron is lower than that of G2-18C. However, this was not the case. Only ≈40 contacts for G0 were establish with siRNA, less than the G1 and G2 systems. This evidence supports the idea that G2-18C spherical micelles can provide nucleic acid a higher contact surface area, which could favour its packaging.

Furthermore, the ratio between the surface area and the total number of amines was estimated for each micelle (Table 3). With this information, the area required by one amine to bind to siRNA can be estimated. The results showed that amines from spherical micelles occupied a larger surface area than those from the G0 rod. The propensity of the G0-18C dendrimer towards rod formation could cause amine group structures to occupy a smaller surface, creating a less efficient aggregate. We hypothesise that in spherical aggregates, such as in G2-18C system, amine groups are distributed across a larger area, thus favouring the neutralization and packing of siRNA.

## Conclusion

This study explored the self-assembly process of PAMAM ADs with different alkyl chains and generations. Molecular dynamics simulations detected that hydrophobic tail length and hydrophilic head size, or dendron generation, had critical impacts on the size, shape, and self-organization of the aggregates, as well as on interactions with nucleic acids.

Research by Yu *et al*.^16^ indicates that G2-18C dendrimers can be successfully used as siRNA carriers, in contrast to dendrimers of the same generation but with shorter alkyl tails and to smaller generation dendrimers with the same tail length. With this experimental evidence in mind, the present study corroborated the self-assembly dynamics of G2-18C dendrimer generated micelles whose size and shape formed the most efficiently organized structures within each amine group. This efficiency assured more effective siRNA transfection in terms of amine-phosphate interactions. Moreover, spherical micelles can effectively package siRNA through beads-on-string structures^47^. In contrast, rod micelles formed by G0 dendrimers can form a packed matrix that could immobilize siRNA molecules and creates a more rigid structure, which could eventually affect cell uptake.

These data contribute to a better atomic understanding of this class of dendrimers and corroborates previous insights about long alkyl chains in amphiphiles favour siRNA packing and increasing complex stability^8^.

## Additional Information

**How to cite this article**: Márquez-Miranda, V. *et al*. Self-Assembly of Amphiphilic Dendrimers: The Role of Generation and Alkyl Chain Length in siRNA Interaction. *Sci. Rep.* **6**, 29436; doi: 10.1038/srep29436 (2016).

## Supplementary Material

Supplementary Information

## Figures and Tables

**Table 1:** Summary of MARTINI CG bead types used to build the siRNA model.

siRNA component	Number of particles	Particle type		
phosphate	1	Qa		
ribose sugar	1	P1		
adenine	3	Na	Na	Nda
uridine	2	Na	Na	
cytosine	2	Na	Nd	
guanine	3	Nda	Nda	Na

**Table 2:** Number of ADs (aggregation number), micelle radius (nm), core radius (nm), alkyl tail length (nm), packing parameter (P), and micelle surface area (nm^2^) for the micelles obtained from G2-18C, G2-15C, and G2-13C systems.

No. ADs	MicelleRadius (nm)	Core Radius (nm)	Tail Length (nm)	Micelle Surface Area (nm^2^)	Core Volume (nm^3^)	Headgroup Volume (nm^3^)	Packing Parameter
G2-18C ADs							
13 *	1.8	1.0	1.2	40.7	4.2	20.2	0.27
18	2.0	1.1	1.7	50.7	6.1	27.9	0.21
19 (1) *	2.1	1.1	1.6	53.4	6.1	30.6	0.23
19 (2)	2.1	1.2	1.7	53.8	6.4	30.7	0.22
24 (1)	2.2	1.2	1.7	60.2	7.7	36.3	0.23
24 (2) *	2.2	1.2	1.7	62.3	7.7	38.5	0.24
26 *	2.2	1.3	1.7	63.6	8.5	39.2	0.24
32	2.4	1.3	1.6	72.6	10.2	48.0	0.28
*Mean*	***2.1***	***1.2***	***1.6***	***57.2***	***7.1***	***33.9***	***0.24***
G2-15C ADs							
15 *	2.0	1.0	1.5	47.9	4.5	26.7	0.22
18	2.0	1.1	1.5	52.6	5.9	30.0	0.24
19(1) *	2.0	1.1	1.6	52.6	5.5	30.3	0.23
19(2) *	2.1	1.1	1.4	52.9	5.9	30.3	0.26
20	2.1	1.1	1.5	53.6	6.1	30.8	0.25
26	2.2	1.2	1.5	63.4	8.0	39.4	0.27
27 *	2.3	1.3	1.5	68.4	8.8	44.4	0.28
31	2.4	1.3	1.5	70.4	9.1	46.5	0.29
*Mean*	***2.1***	***1.2***	***1.5***	***57.7***	***6.7***	***34.8***	***0.26***
G2-13C ADs							
12 *	1.8	0.9	1.2	39.0	3.2	19.7	0.25
13 *	1.9	0.9	1.2	43.3	3.4	23.4	0.25
15 *	1.9	1.0	1.1	44.6	4.3	23.7	0.31
19(1)	2.1	1.1	1.3	53.0	5.0	31.2	0.27
19(2)	2.0	1.1	1.3	50.1	4.9	28.4	0.28
22(1)	2.1	1.1	1.2	56.6	5.5	34.5	0.29
22(2)	2.1	1.1	1.2	56.3	5.7	33.9	0.29
24 *	2.2	1.1	1.2	58.1	5.6	36.1	0.3
29	2.4	1.3	1.2	70.8	9.6	46.4	0.35
*Mean*	***2.0***	***1.1***	***1.2***	***52.4***	***5.3***	***30.8***	***0.29***
G1-18C ADs							
28 *	1.9	1.3	1.7	43.9	9.0	18.4	0.25
40 *	2.1	1.4	1.7	53.8	12.7	24.5	0.28
49	2.2	1.6	1.7	62.2	15.6	30.5	0.30
58 *	2.3	1.6	1.6	68.9	18.6	35.2	0.34
*Mean*	***2.1***	***1.5***	***1.7***	***57.2***	***14.0***	***27.1***	***0.29***
G0-18C ADs							
18	1.4	1.1	1.5	23.6	5.5	5.3	0.24
26 *	1.5	1.2	1.7	29.5	8.1	6.9	0.25
131 *	3.1	2.6	1.7	77.4	48.8	63.9	0.61
	3.8	3.1					
	2.3	1.5					
*Mean*	***2.4***	***1.9***	***1.6***	***43.5***	***20.8***	***25.4***	***0.36***

Micelles that are effectively bound to siRNA are indicated with an asterisk (*).

**Table 3:** Number of amines per micelle, mean number of amines per micelle bound to siRNA, and surface area occupied by a single amine group.

System	#ADs per micelle	Total #Amines per micelle	Mean#Amines binding siRNA	Surface area per amine grougs (nm^2^)
G2-18C	19	152	21.7 ± 1.6	0.35
G2-18C	26	208	25.5 ± 1.3	0.35
G2-18C	24	192	13.6 ± 1.2	0.32
G2-18C	13	96	15.0 ± 1.6	0.42
G2-15C	19 (1)	152	19.9 ± 1.6	0.35
G2-15C	19 (2)	152	23.7 ± 1.5	0.35
G2-15C	15	120	20.7 ± 1.6	0.40
G2-15C	27	216	6.3 ± 2.6	0.31
G2-13C	12	96	15.8 ± 2.0	0.40
G2-13C	13	104	18.8 ± 1.6	0.41
G2-13C	15	120	16.1 ± 2.0	0.37
G2-13C	24	192	18.2 ± 1.9	0.30
G1-18C	28	112	22.9 ± 1.8	0.39
G1-18C	40	160	19.2 ± 1.8	0.33
G1-18C	58	232	24.4 ± 1.9	0.29
G0-18C	26	52	8.6 ± 1.3	0.56
G0-18C	131	262	30.7 ± 1.9	0.29
