# Supplementary material for: Self-Assembly of Amphiphilic Dendrimers: The Role of Generation and Alkyl Chain Length in siRNA Interaction
Source: Sci Rep. 2016 Jul 5;6:29436. doi: 10.1038/srep29436 (PMC4932498; doi:10.1038/srep29436)
Supplement: Supplementary Information [file srep29436-s1.pdf]

Supporting Information

Self-Assembly of Amphiphilic Dendrimers: The Role of Generation and Alkyl Chain Length in siRNA Interaction

Valeria Márquez-Miranda,[†,‡] *Ingrid Araya-Durán,*[†,‡] *María Belén Camarada,* [§] *Jeffrey Comer,*[¶] *Jesús A. Valencia-Gallegos,*[¥] *Fernando Danilo González-Nilo,**[†,‡,]

[†]Universidad Andres Bello, Facultad de Biología, Center for Bioinformatics and Integrative Biology (CBIB), Av. República 239, Santiago, Chile; [‡]Fundación Fraunhofer Chile Research, Las Condes, Chile; [§] Universidad Bernardo O Higgins, Laboratorio de Bionanotecnología, General Gana 1702, Santiago, Chile; *Centro Interdisciplinario de Neurociencia de Valparaíso, Facultad de Ciencias, Universidad de Valparaíso, Valparaíso, Chile; [¶]Kansas State University, Nanotechnology Innovation Center of Kansas State, Institute of Computational Comparative Medicine, Anatomy and Physiology; and [¥]Centro de Biotecnología FEMSA, Escuela de Ingeniería y Ciencias, Tecnológico de Monterrey, Av. Eugenio Garza Sada 2501 Sur, Col. Tecnológico, Monterrey, N.L, México.

Corresponding Author

*E-mail: fernando.gonzalez@unab.cl.

Full atom Molecular Simulations of Amphiphilic Dendrimers.

Full atomistic simulations of single ADs in explicit water were transformed to CG resolution using the mapping technique described in Figure 1. Molecular dynamics were run using an isobaric-isothermal (NPT) ensemble, where the number of particles, the pressure and the temperature were held constant. As AD's structures are not included in conventional force fields, each system was parameterized. Parameters and topology for each AD were obtained from CHARMM General Force Field (CGenFF)[1] and PARAMCHEM website (http://www.paramchem.org)[2,3] in accordance with CHARMM philosophy. Dihedrals and angles were adjusted in order to fit geometries optimized at Density Functional Theory level (B3LYP/6-311G[4-6]) using the computational package Gaussian09 [7]. Systems underwent 2000 steps of energy minimization followed by 20 ns of dynamics. A Langevin thermostat was employed to keep a mean temperature of 310 K and Langevin piston[8] method was considered to maintain a mean pressure of 1 atm. All molecular dynamics simulations were performed using the computational code NAMD[9]. The equations of motion were integrated with a 2.0 fs time step along with SETTLE[10] and RATTLE[11] to constrain the geometry of the water molecules and the length of covalent bonds to hydrogen atoms. Non-bonded energies were calculated using particle-mesh Ewald full electrostatics[12] (grid spacing < 0:10 nm). Long-range electrostatic interactions were calculated every 2 steps using a multiple-time stepping scheme. Interactions among AD's and TIP3P water molecules[13] were computed using the CHARMM27 force field[14,15]. All simulation systems were cubic in shape, and periodic boundary conditions were enforced in all three directions. A 0.15 M concentration of Na_+ and Cl^- ions was added to each simulation box.

In order to validate the CG scheme, the variation of the radius of gyration was monitored for each AD and compared to the average value obtained by full atomistic simulations.

References

[1] K. Vanommeslaeghe, E. Hatcher, C. Acharya, S. Kundu, S. Zhong, J. Shim, E. Darian, O. Guvench, P. Lopes, I. Vorobyov, A.D. Mackerell, Journal of Computational Chemistry 31 (2010) 671.

[2] K. Vanommeslaeghe, A.D. MacKerell, Journal of Chemical Information and Modeling 52 (2012) 3144.

[3] K. Vanommeslaeghe, E.P. Raman, A.D. MacKerell, Journal of Chemical Information and Modeling 52 (2012) 3155.

[4] A.D. Becke, J. Chem. Phys 98 (1993) 5648.

[5] C. Lee, W. Yang, R.G. Parr, Phys. Rev. B 37 (1988) 785.

[6] P.J. Stephens, F.J. Devlin, C.F. Chabalowski, M.J. Frisch, J. Phys. Chem. 98 (1994) 11623.

[7] M.J. Frisch, G.W. Trucks, H.B. Schlegel, G.E. Scuseria, M.A. Robb, J.R. Cheeseman, G. Scalmani, V. Barone, B. Mennucci, G.A. Petersson, H. Nakatsuji, M. Caricato, X. Li, H.P. Hratchian, A.F. Izmaylov, J. Bloino, G. Zheng, J.L. Sonnenberg, M. Hada, M. Ehara, K. Toyota, R. Fukuda, J. Hasegawa, M. Ishida, T. Nakajima, Y. Honda, O. Kitao, H. Nakai, T. Vreven, J.A. Montgomery, J.E. Peralta, F. Ogliaro, M. Bearpark, J.J. Heyd, E. Brothers, K.N. Kudin, V.N. Staroverov, R. Kobayashi, J. Normand, K. Raghavachari, A. Rendell, J.C. Burant, S.S. Iyengar, J. Tomasi, M. Cossi, N. Rega, J.M. Millam, M. Klene,

J.E. Knox, J.B. Cross, V. Bakken, C. Adamo, J. Jaramillo, R. Gomperts, R.E. Stratmann, O. Yazyev, A.J. Austin, R. Cammi, C. Pomelli, J.W. Ochterski, R.L. Martin, K. Morokuma, V.G. Zakrzewski, G.A. Voth, P. Salvador, J.J. Dannenberg, S. Dapprich, A.D. Daniels, Farkas, J.B. Foresman, J.V. Ortiz, J. Cioslowski, D.J. Fox. Gaussian 2009.
[8] G.J. Martyna, D.J. Tobias, M.L. Klein, J. Chem. Phys. 101 (1994) 4177.
[9] J.C. Phillips, R. Braun, W. Wang, J. Gumbart, E. Tajkhorshid, E. Villa, C. Chipot, R.D. Skeel, L. Kalé, K. Schulten, Journal of Computational Chemistry 26 (2005) 1781.
[10] S. Miyamoto, P.A. Kollman, J. Comput. Chem. 13 (1992) 952.
[11] H.J. Andersen, Comp. Phys. 52 (1983) 24.
[12] T. Darden, D. York, L.J. Pedersen, Chem. Phys. Lett. 98 (1993) 10089.
[13] W.L. Jorgensen, J. Chandrasekhar, J.D. Madura, R.W. Impey, M.L. Klein, The Journal of Chemical Physics 79 (1983) 926.
[14] J. MacKerell, A. D. , D. Bashford, M. Bellott, R.L. Dunbrack Jr., J.D. Evanseck, M.J. Field, S. Fischer, J. Gao, H. Guo, S. Ha, D. Joseph-McCarthy, L. Kuchnir, K. Kuczera, F.T.K. Lau, C. Mattos, S. Michnick, T. Ngo, D.T. Nguyen, B. Prodhom, I. Reiher, W.E., B. Roux, M. Schlenkrich, J.C. Smith, R. Stote, J. Straub, M. Watanabe, J. Wiorkiewicz-Kuczera, D. Yin, M. Karplus, Journal of Physical Chemistry B 102 (1998) 3586.
[15] D. Beglov, B. Roux, Journal of Chemical Physics 100 (1994) 9050.

Supporting Table S1. Number of micelles and amphiphilic dendrimers (ADs) per micelle, $R_{micelle}$(nm), R_{core} (nm), and R_{shell} (nm) in an 18-Carbon tail length AD systems, in absence of siRNA.

Number of micelles	AD per micelle	$R_{micelle}$ (nm)	R_{core} (nm)	R_{shell} (nm)
		**G2-18C**		
**1**	6	1.5	0.8	0.7
**1**	15	1.9	1.1	0.9
**1**	16	2.0	1.1	0.9
**1**	19	2.1	1.1	0.9
**2**	22	2.2	1.2	1.0
		**G2-15C**		
**1**	12	1.8	1.0	0.9
**1**	13	1.9	1.0	0.8
**1**	16	1.9	1.0	0.9
**2**	19	2.1	1.1	1.0
**1**	21	2.1	1.2	1.0
		**G2-13C**		
**1**	4	1.3	0.6	0.7
**1**	11	1.7	0.9	0.8
**1**	13	1.8	0.9	0.9
**1**	15	1.9	1.0	0.9
**2**	8	1.6	0.8	0.8
**1**	17	2.0	1.0	1.0
**1**	24	2.2	1.2	1.0

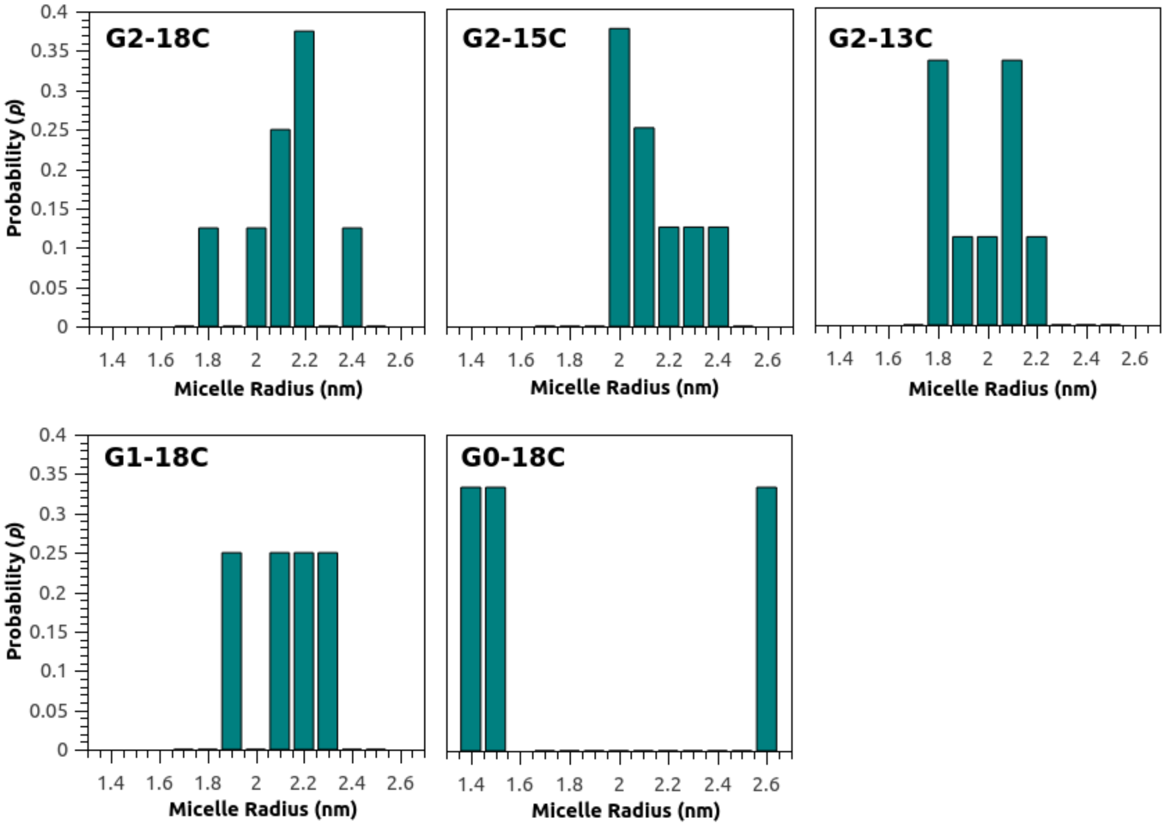

Supporting Figure S1. Distribution of micelle radius R_m in each system.

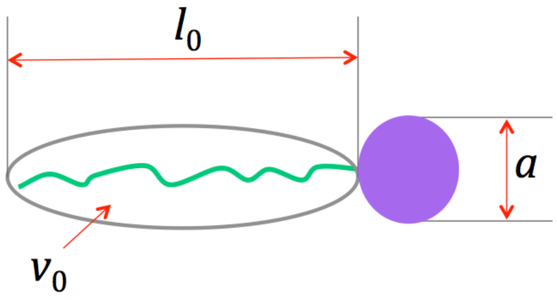

Supporting Figure S2. Representation of amphiphilic dendrimer geometry, where v_0 is the volume of the hydrophobic chain; a is the surface area of the hydrophobic core of the aggregate, expressed per aggregate molecule (i.e. the area per molecule); and l_0 is the critical length of the hydrophobic tail.

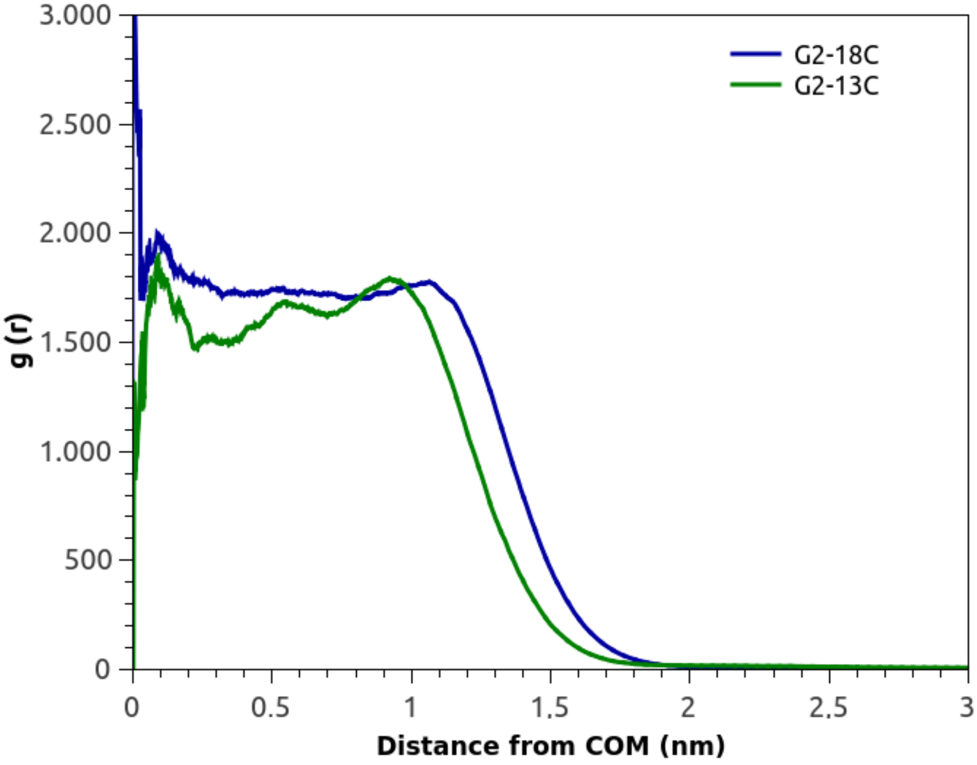

Supporting Figure S3. Radial distribution function of the hydrophobic tails of two 19 amphiphilic dendrimer (AD) micelles from the G2-18C and G2-13C systems.

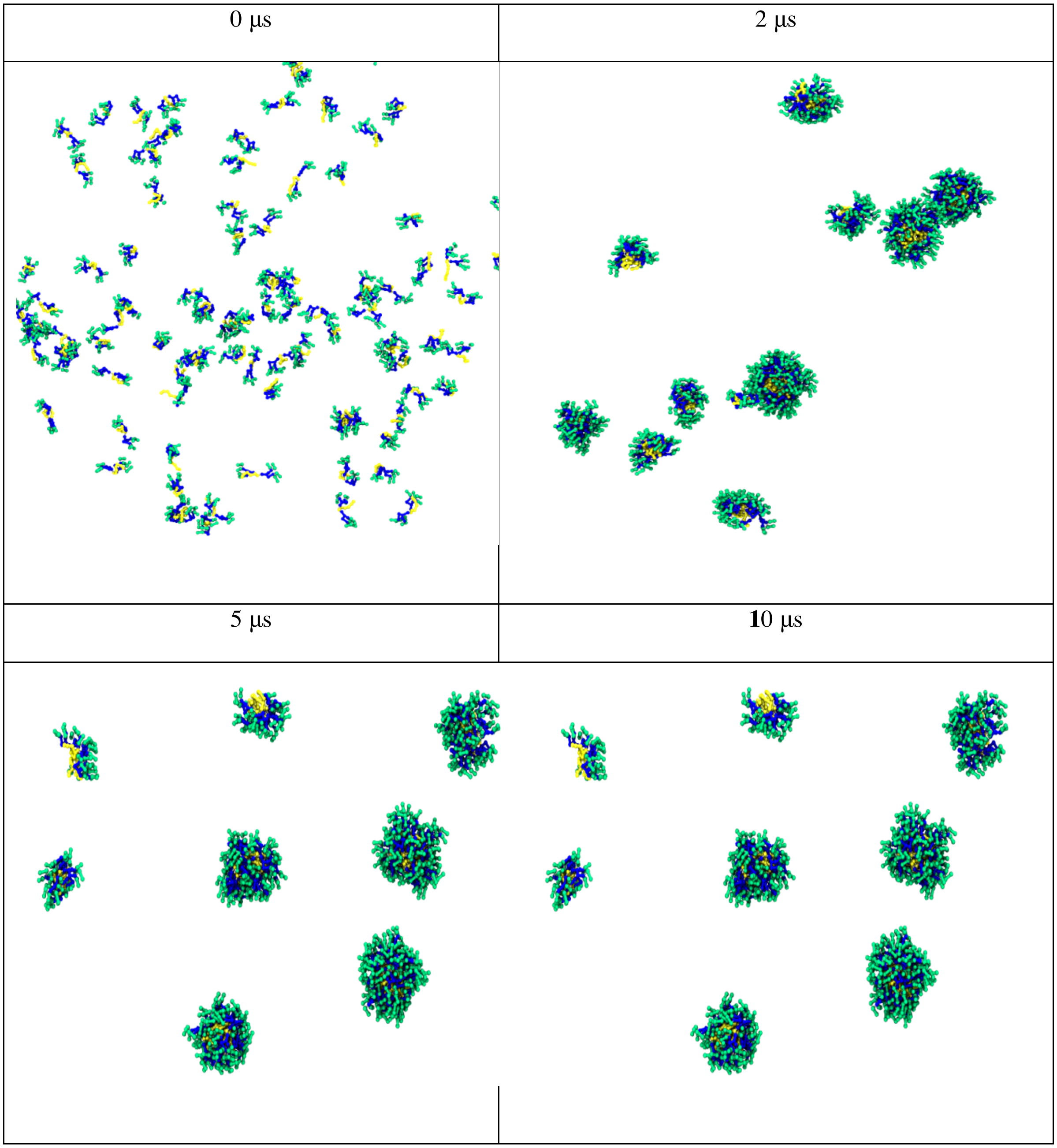

Supporting Figure S4. Self-assembly of G2-18C dendrimers in solution in the absence of a siRNA molecule. Four snapshots were taken at 0 μs, 2 μs, 5 μs, and 10 μs of molecular dynamics trajectory.

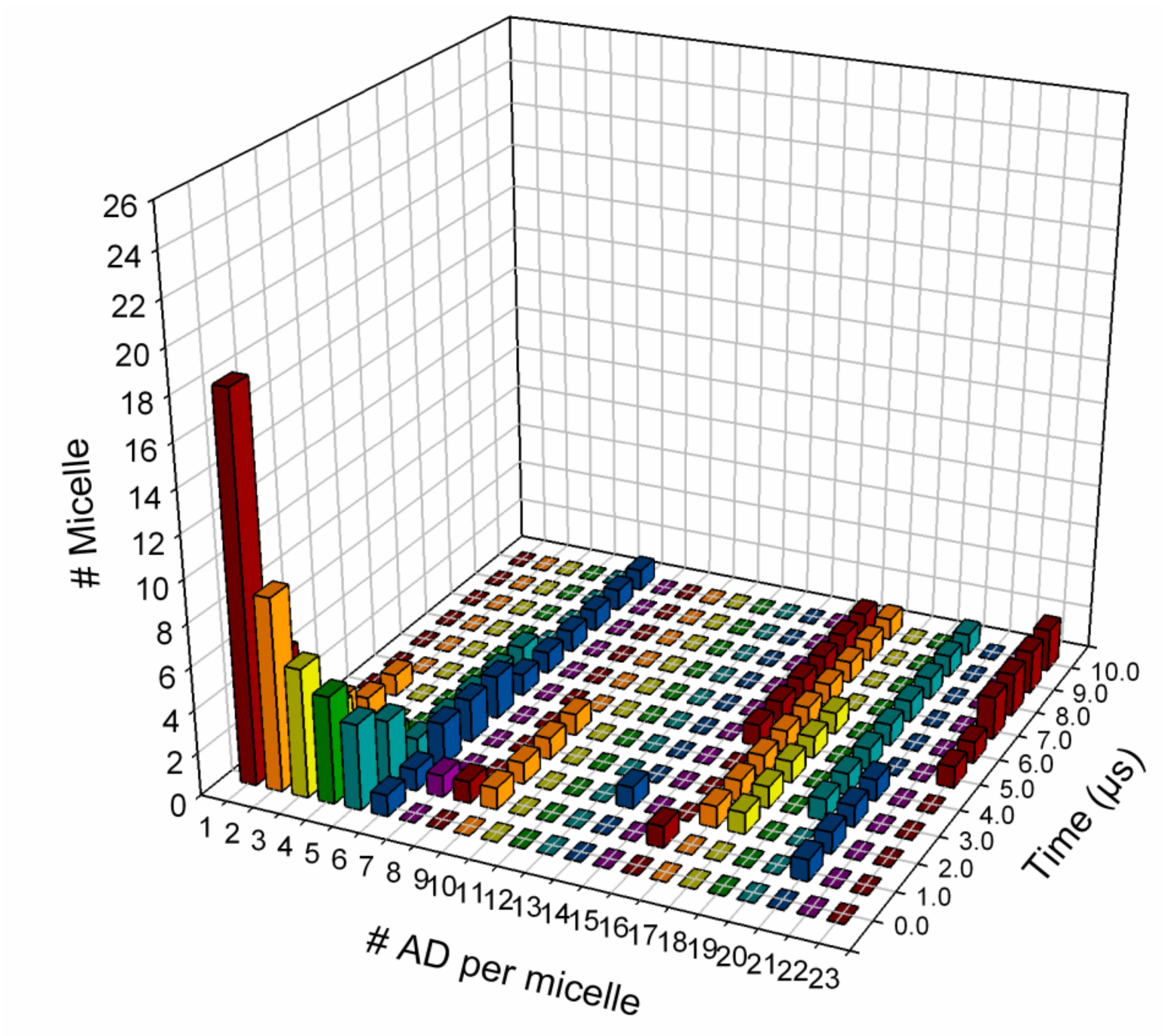

Supporting Figure S5. Number of aggregates (micelles) as a function of simulation time for G2-18C amphiphilic dendrimers (ADs) in the absence of siRNA. A molar concentration of 3 mM of G2-18C ADs was considered. At the beginning of the simulation, there were a high number of aggregates with few ADs. As the simulation continued, the number of micelles with a higher number of ADs increased. At the end of the simulation, two aggregates of 22 ADs were detected.

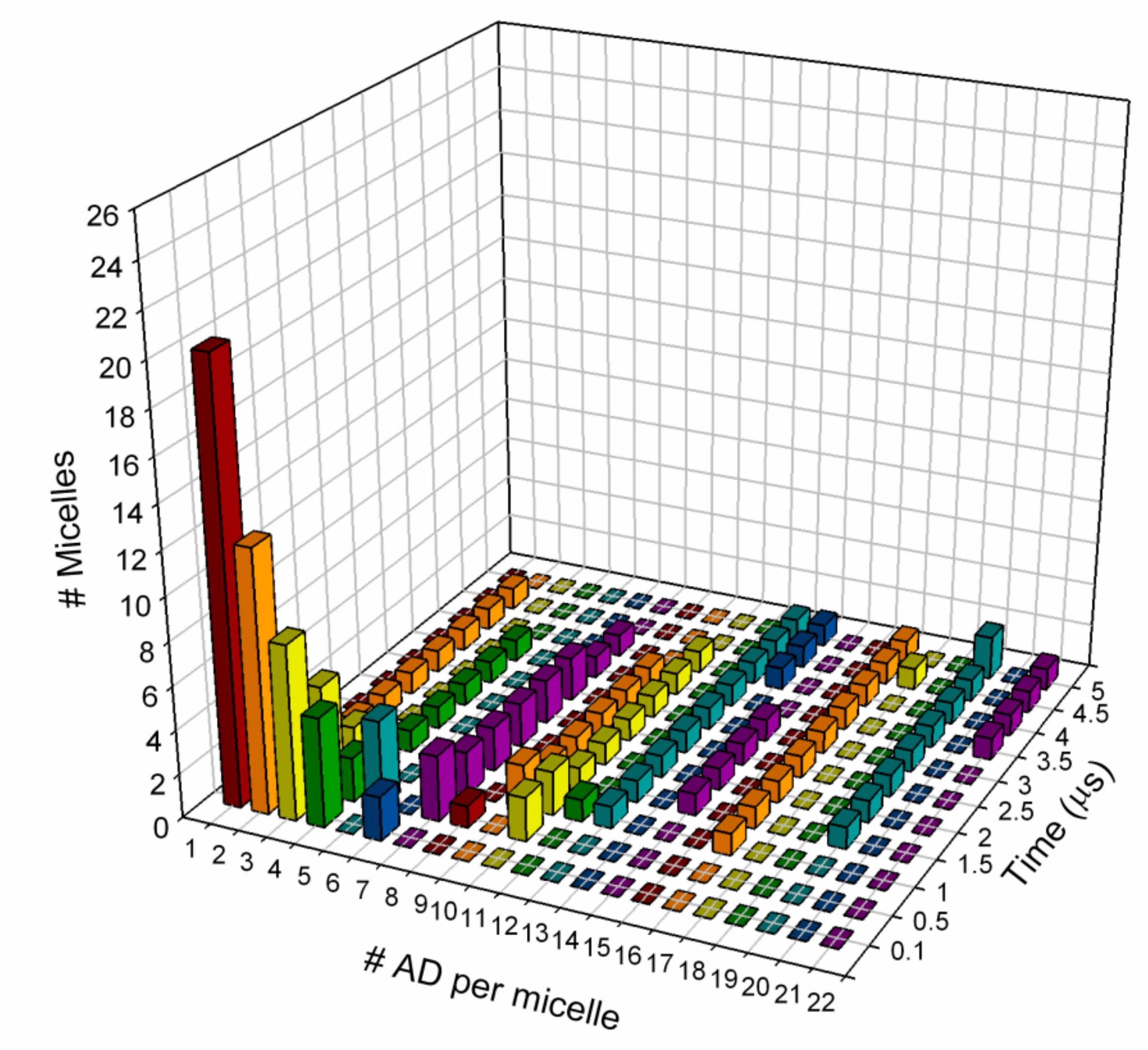

Supporting Figure S6. Number of aggregates (micelles) as a function of simulation time for G2-15C amphiphilic dendrimers (ADs) in the absence of siRNA. A molar concentration of 3 mM of G2-15C dendrimers was considered. At the beginning of the simulation, there were a high number of aggregates with few ADs. As the simulation continued, the number of micelles with a higher number of ADs increased. Micelles of 12, 13, 16, 19, and 22 ADs remained until the end of the simulation.

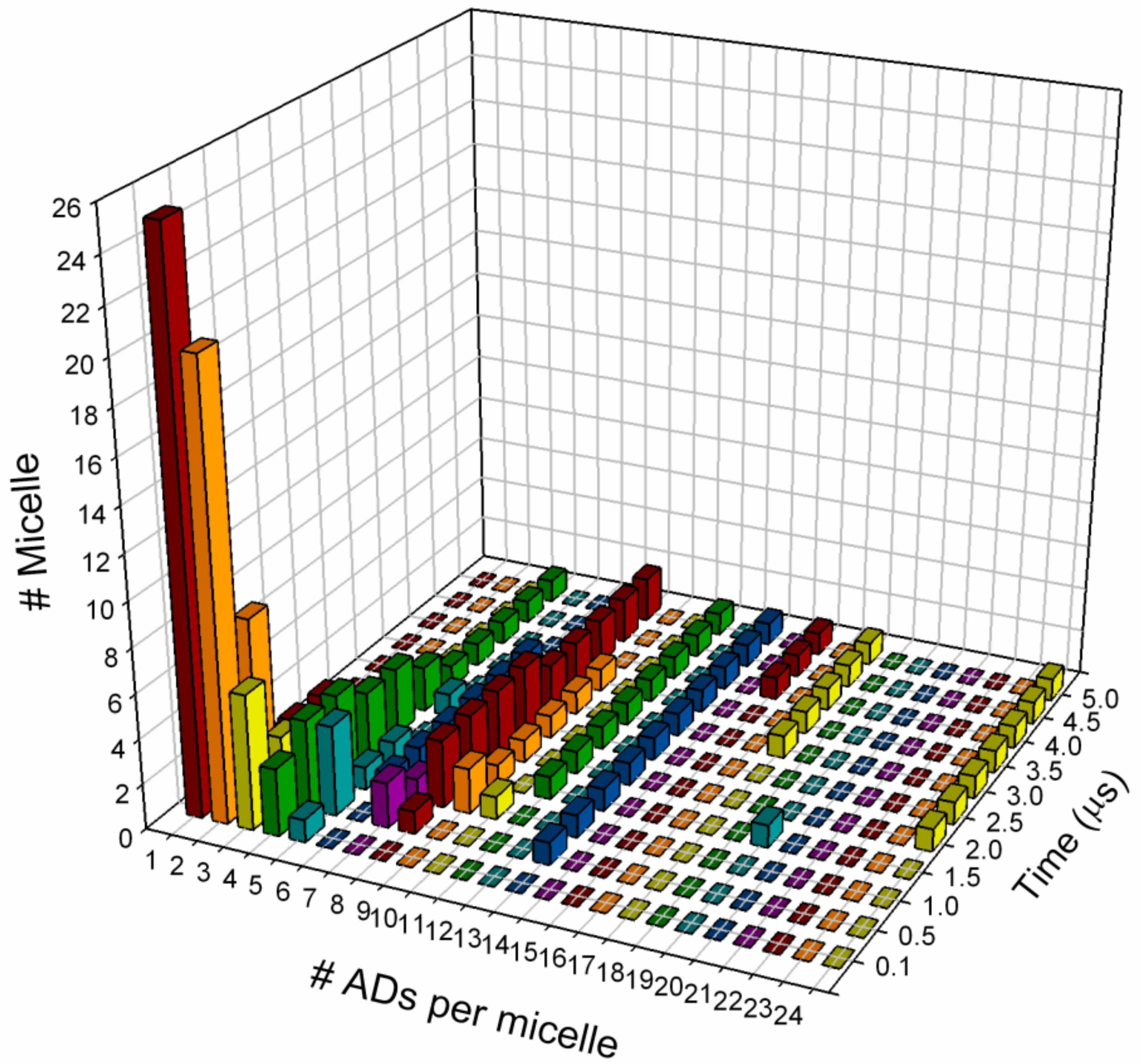

Supporting Figure S7. Number of aggregates (micelles) as a function of simulation time for G2-13C amphiphilic dendrimers (ADs) in the absence of siRNA. A molar concentration of 3 mM of G2-13C dendrimers was considered. At the beginning of the simulation, there were a high number of aggregates with few ADs. As the simulation continued, the number of micelles with a higher number of ADs increased. Micelles of 4, 8, 11, 17, and 24 ADs remained until the end of the simulation.

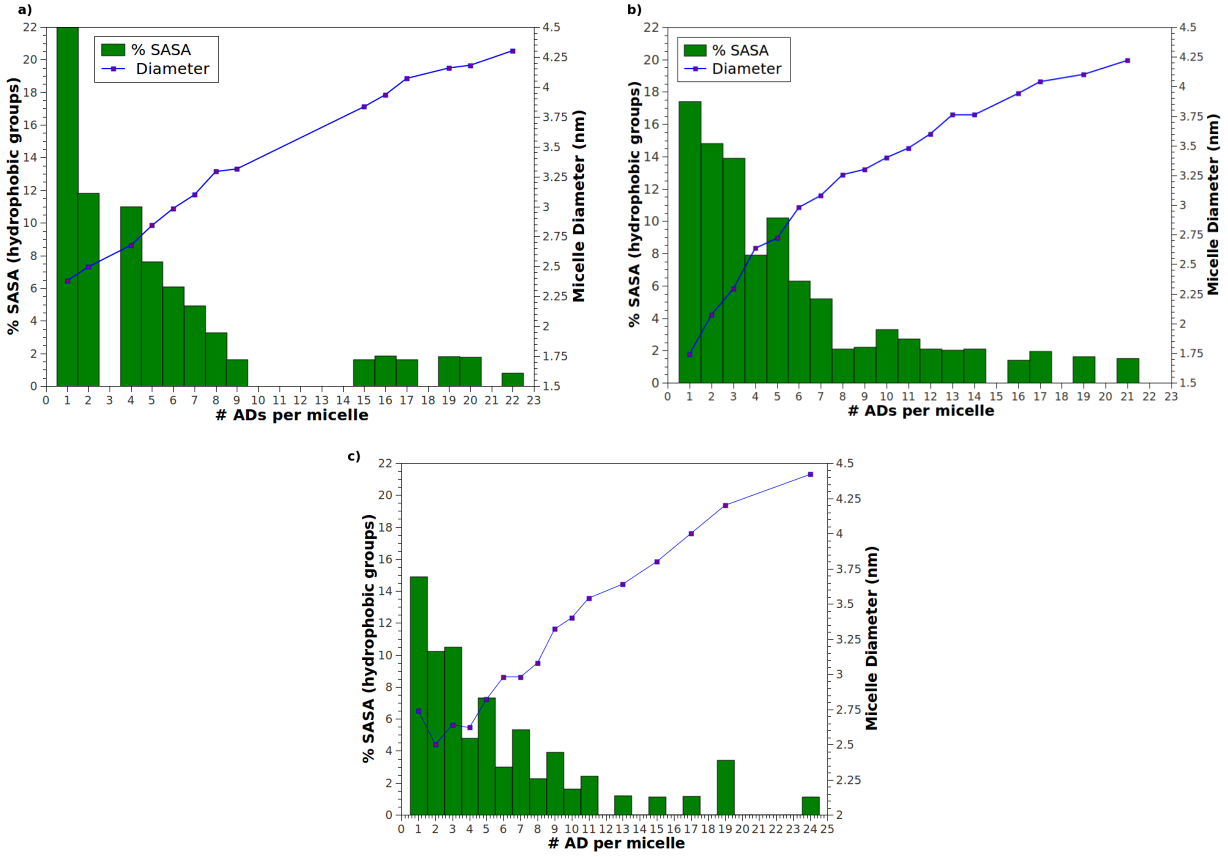

Supporting Figure S8. Percentage of Solvent Accessible Surface Area (%SASA) of hydrophobic tails (compared to total SASA of the micelles) and micelle diameter (nm) as a function of the number of amphiphilic dendrimers (ADs) per micelle for (a) G2-18C, (b) G2-15C, and (c) G2-13C. As the number of ADs per micelle increased, micelle diameter linearly increased. Meanwhile, the %SASA decreased until plateauing when the micelle was composed of ≈ 15 ADs.

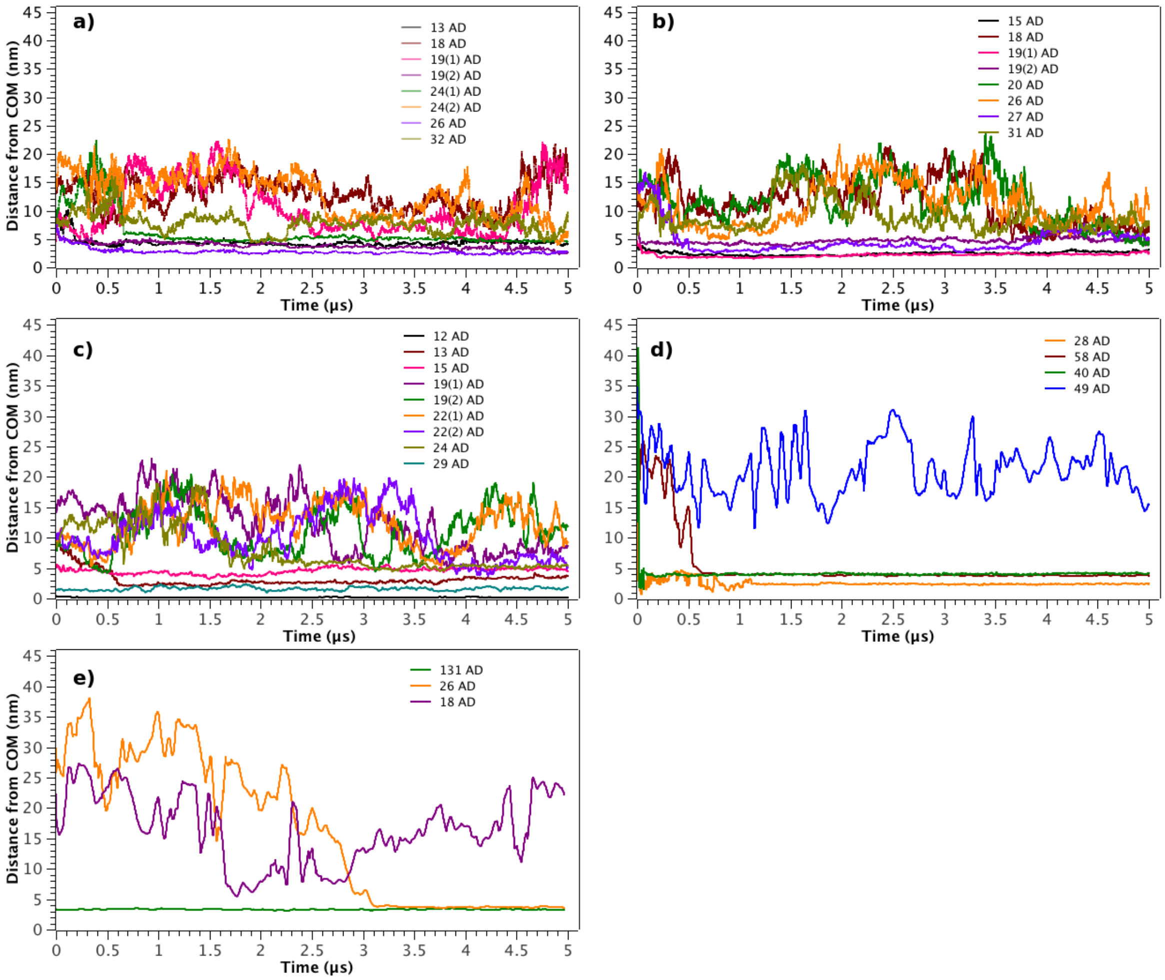

Supporting Figure S9. Centre-of-mass (COM) distance between each micelle and the siRNA fragment in (a) G2-18C, (b) G2-15C, (c) G2-13C, (d) G1-18C, and (e) G0-18C systems. In systems with G2 dendrons, four micelles were found close to siRNA (< 7 nm) and remained stable throughout the whole simulation, forming a cluster. The other micelles tended to agglomerate close to the cluster**.** Only three (G1-18C) and two (G0-18C) micelles were able to form a complex with siRNA.

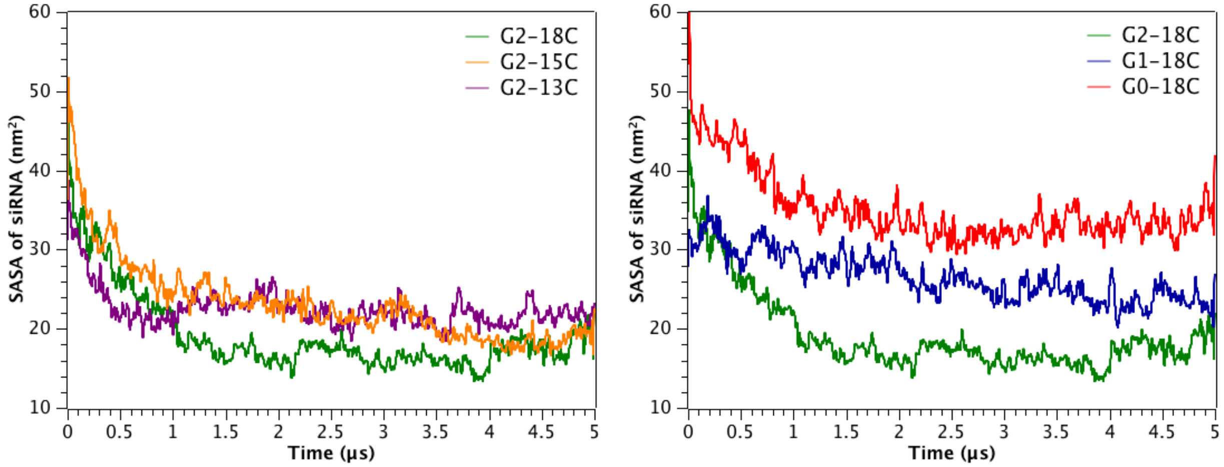

Supporting Figure S10 . SASA of siRNA in the presence of dendrimers (a) G2-18C, G2-15C, and G2-13C; and (b) G2-18C, G1-18C, and G0-18C, taken from Replica #2.

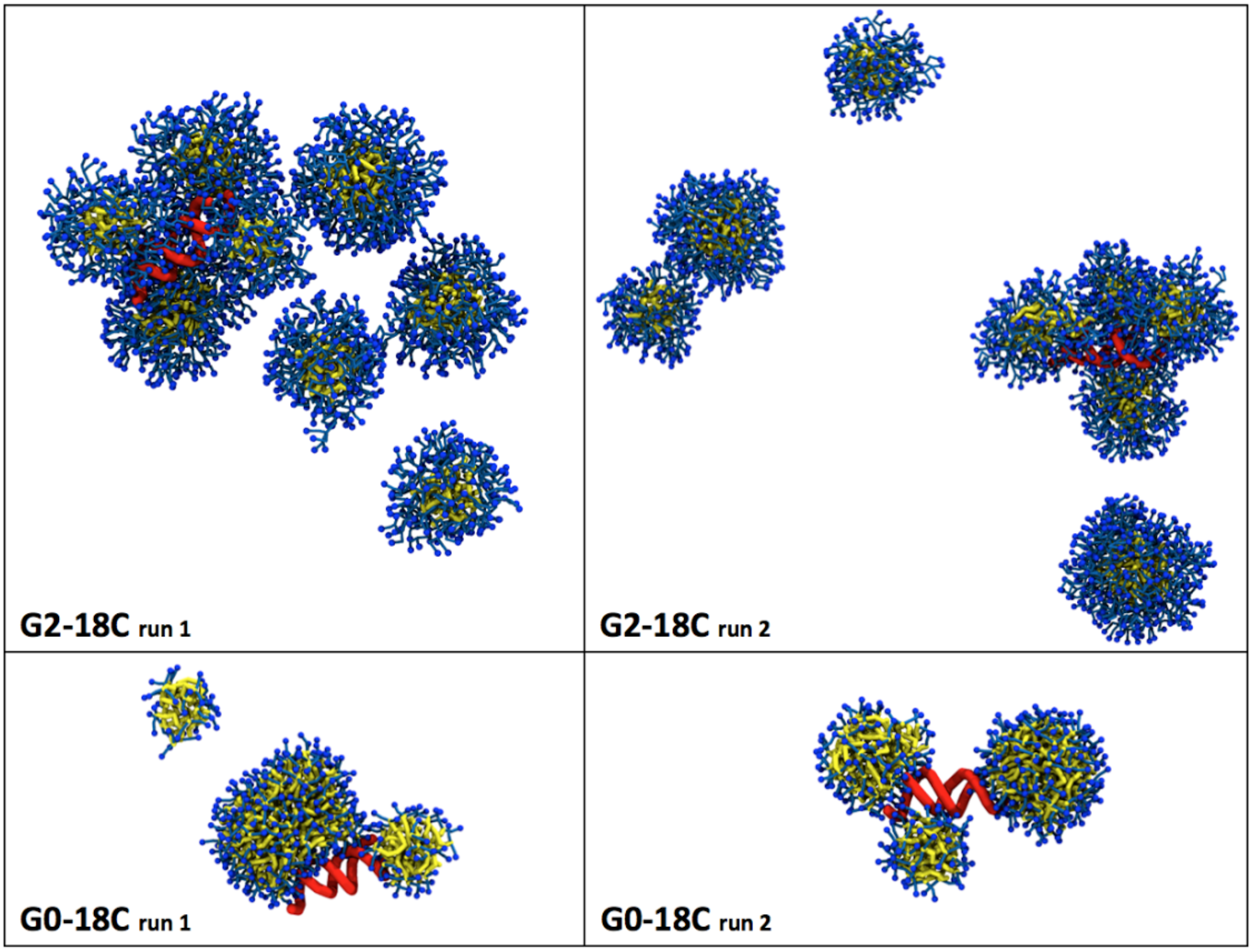

Supporting Figure S11. Last snapshot of MD simulations (Run #1 and Run #2) of G2-18C and G0-18C systems.

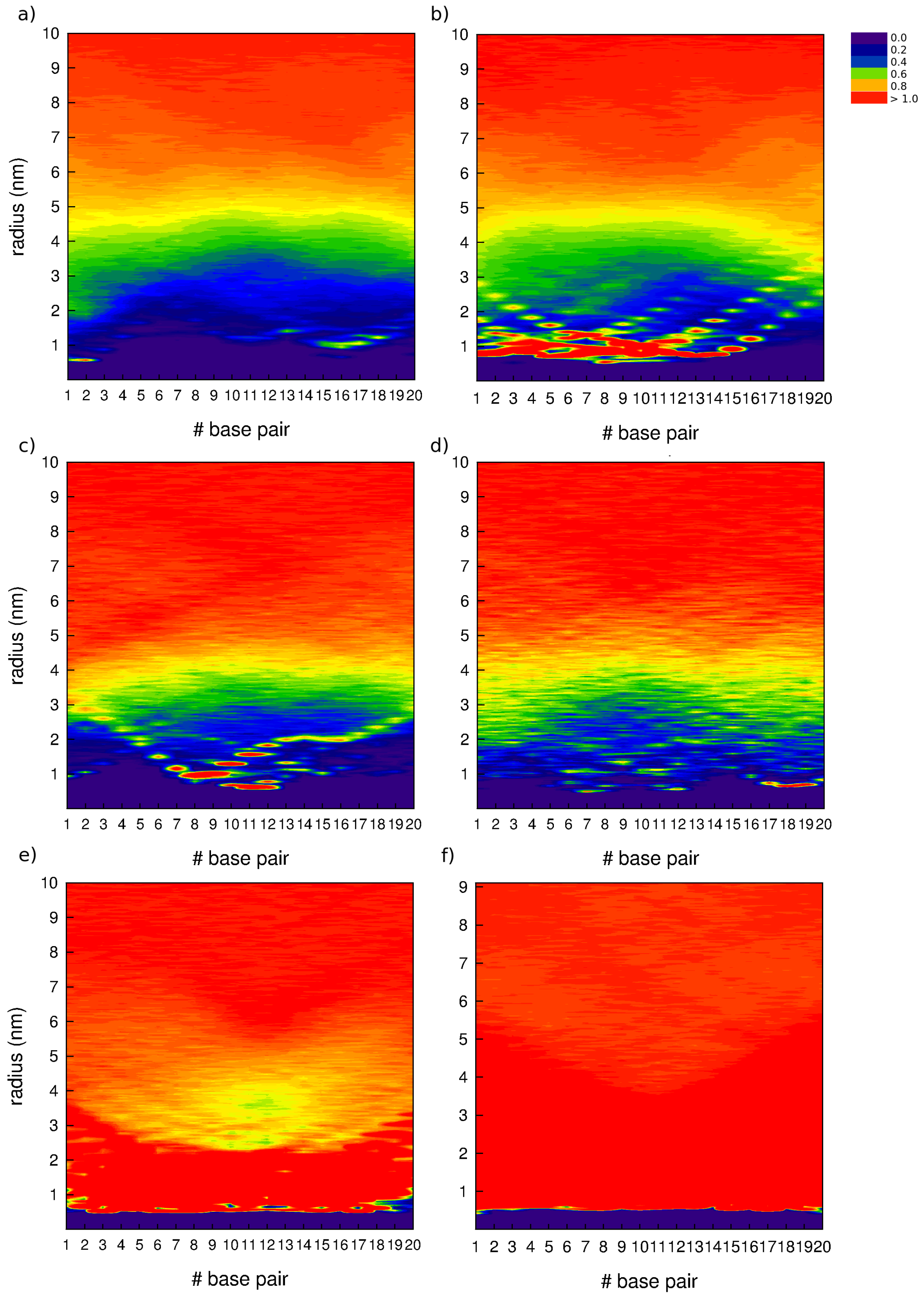

Supporting Figure S12. Radial distribution function (RDF) of sodium counterions as a function of the distance from each siRNA base-pair centre-of-mass (COM), for each system (a) G2-18C, (b) G2-15C, (c) G2-

13C, (d) G1-18C, (e) G0-18C. Data of the distribution of sodium in a system containing only siRNA have been added as a reference (f). Each base pair is represented in the x axis, radius (nm) in the y axis and surface in colours (z) represents the RDF. A small fringe along the x axis, where y< 0.5 nm (purple), represents the zone where siRNA atoms are present.

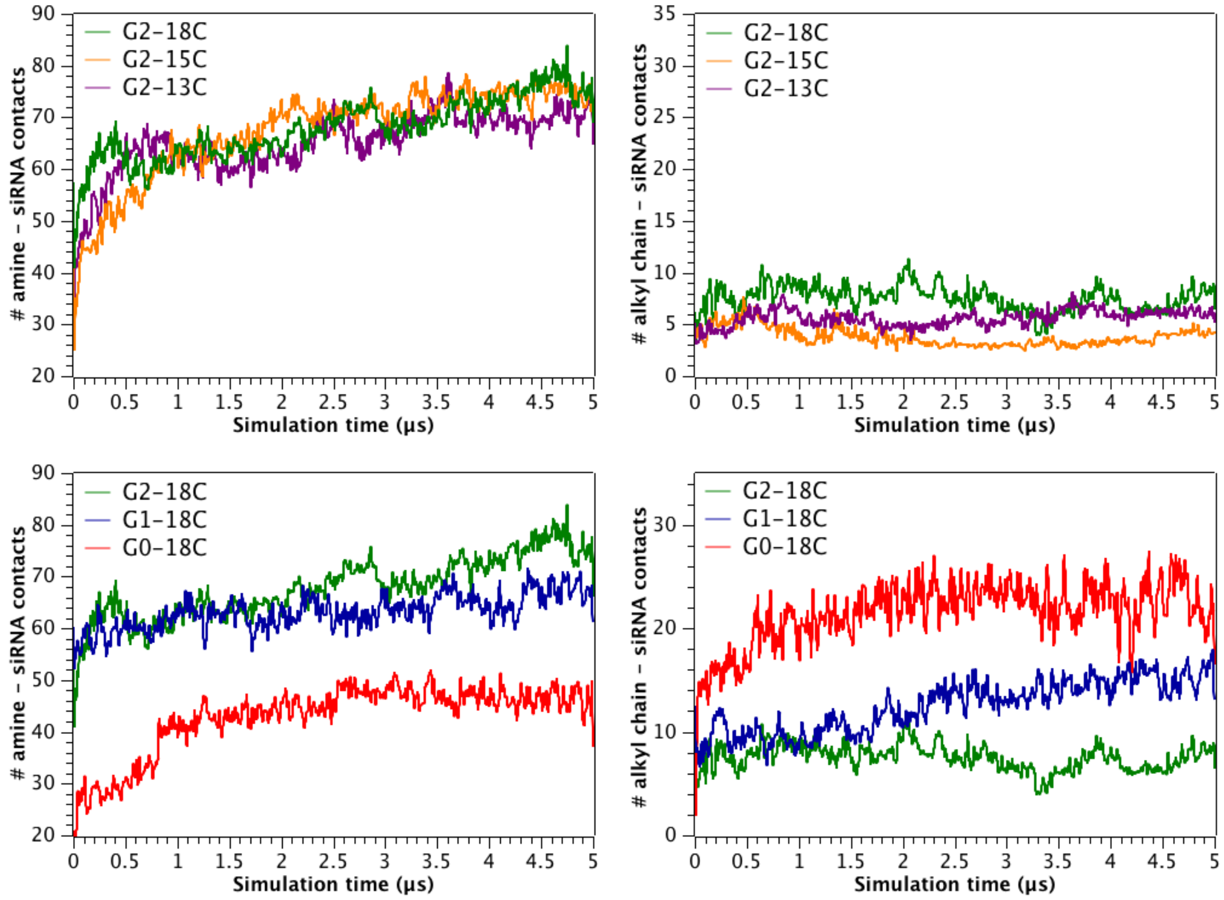

Supporting Figure S13. Time evolution for the number of contacts between amine groups of dendrimers and siRNA at 0.75 nm and alkyl chains and siRNA at 0.75 nm. (a) and (b) represent data for G2-18C, G2-15C, G2-13C and (c) and (d) for G2-18C, G1-18C, G0-18C, Data were taken from Replica #2.

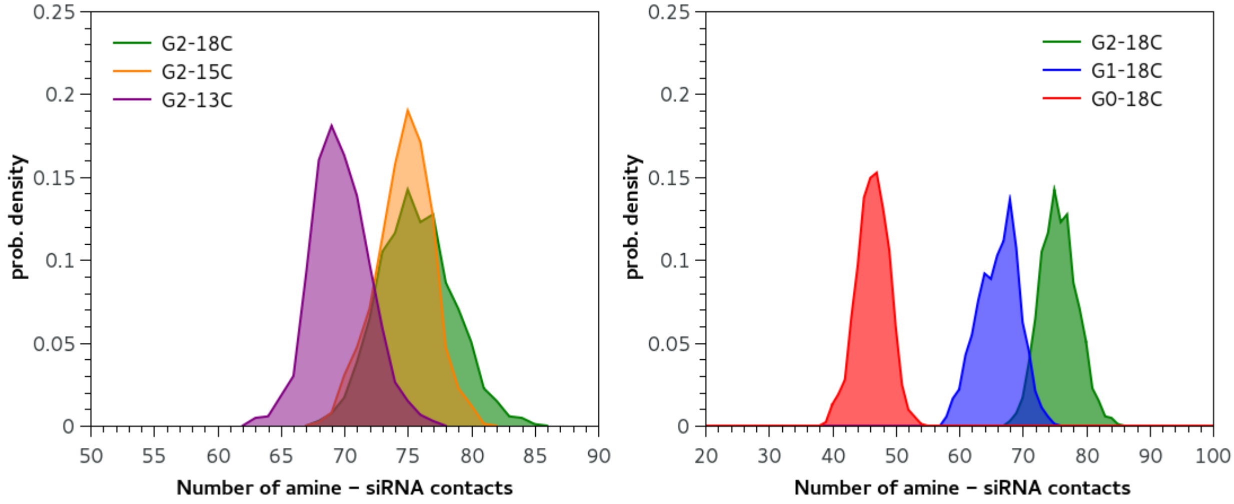

Supporting Figure S14. Histogram of the number of contacts between amine-terminal groups of the dendrimers and phosphate groups of siRNA, for a) G2-18C, G2-15C, G2-13C and b) G2-18C, G1-18C, G0-18C. Density was normalized considering the total values recorded for the last microsecond of simulation. Data were taken from Replica #2.
